# Mechanistic details of CRISPR-associated transposon recruitment and integration revealed by cryo-EM

**DOI:** 10.1073/pnas.2202590119

**Published:** 2022-08-01

**Authors:** Jung-Un Park, Amy Wei-Lun Tsai, Tiffany H. Chen, Joseph E. Peters, Elizabeth H. Kellogg

**Affiliations:** ^a^Department of Molecular Biology and Genetics, Cornell University, Ithaca, NY 14853;; ^b^Department of Microbiology, Cornell University, Ithaca, NY 14853

**Keywords:** CRISPR-associated transposon, cryo-EM, TnsB, transposase structure

## Abstract

CRISPR-associated transposons (CASTs) are Tn7-like elements that are capable of RNA-guided DNA integration. Although structural data are known for nearly all core transposition components, the transposase component, TnsB, remains uncharacterized. Using cryo-electron microscopy (cryo-EM) structure determination, we reveal the conformation of TnsB during transposon integration for the type V-K CAST system from *Scytonema hofmanni* (ShCAST). Our structure of TnsB is a tetramer, revealing strong mechanistic relationships with the overall architecture of RNaseH transposases/integrases in general, and in particular the MuA transposase from bacteriophage Mu. However, key structural differences in the C-terminal domains indicate that TnsB’s tetrameric architecture is stabilized by a different set of protein–protein interactions compared with MuA. We describe the base-specific interactions along the TnsB binding site, which explain how different CAST elements can function on cognate mobile elements independent of one another. We observe that melting of the 5′ nontransferred strand of the transposon end is a structural feature stabilized by TnsB and furthermore is crucial for donor–DNA integration. Although not observed in the TnsB strand-transfer complex, the C-terminal end of TnsB serves a crucial role in transposase recruitment to the target site. The C-terminal end of TnsB adopts a short, structured 15-residue “hook” that decorates TnsC filaments. Unlike full-length TnsB, C-terminal fragments do not appear to stimulate filament disassembly using two different assays, suggesting that additional interactions between TnsB and TnsC are required for redistributing TnsC to appropriate targets. The structural information presented here will help guide future work in modifying these important systems as programmable gene integration tools.

## Significance

CRISPR-associated transposons (CASTs) show tremendous promise for genome engineering yet remain poorly understood. Here, we present the cryo-electron microscopy structure of the transposase (TnsB) from the V-K CAST element from *Scytonema hofmanni* (ShCAST). We determine the molecular mechanism of TnsB recruitment to the target site (via the AAA+ regulator TnsC) and the structural details of the TnsB transposase. This TnsB structure reveals architectural similarities to MuA, but also key structural differences that are significant for understanding CAST transposition. Importantly, we highlight a base-flipping mechanism for stabilizing the 5′ end of the transposon, potentially to ensure the fidelity of synaptic complex assembly. The structures presented here provide a direct target for rational, structure-guided design strategies and re-engineering of CAST elements.

CRISPR-associated transposons (CASTs) have co-opted Cas genes for RNA-guided DNA integration and are promising candidates for novel genome-editing methods (1, 2). CAST elements are fascinating because of their ability to integrate DNA payloads contained within the element at a precise position, with a specific orientation, and in a programmable manner (3–6). CAST elements are evolutionarily related to Tn7 elements and are often referred to as “Tn7-like” (2). Accordingly, Tn7 and Tn7-like CAST elements contain multiple conserved genes that likely share common functions, leading to newfound appreciation for decades of biochemical, genetic, and structural work on Tn7 and related elements (7, 8).

Despite remarkable diversity (1, 8, 9), all RNA-directed transposition systems characterized to date share multiple components: a CRISPR effector (Cas12k or Cascade), proteins dedicated to target capture (TniQ + TnsC), and a transposase called TnsB. By analogy to work from prototypic Tn7 (2), TnsB carries out transposon end recognition, pairing, and the chemical steps which result in integration of cognate element DNA. The V-K CAST system from *Scytonema hofmanni* (ShCAST) is especially appealing as a model system for mechanistic studies due to its simplicity (a single polypeptide chain encodes the effector) and robust in vitro activity (4). Currently, structural information on components Cas12k (10, 11), TniQ, and TnsC (11, 12) exists except for the TnsB transposase, and it remains mysterious how these indispensable components interact to precisely direct insertions into a guide RNA–directed target site. More generally, structural information is required for the TnsB transposase to obtain a mechanistic understanding of the Tn7 and Tn7-like elements given their broad distribution across diverse bacteria with many interesting targeting modalities, including all of the functionally described CAST elements.

Despite their similarities, the transposase components of the aforementioned transposons do not behave identically, and components are not interchangeable. ShCAST, like bacteriophage Mu, likely uses a replicative transposition mechanism (13) involving host-primed DNA replication of the element to generate cointegrates between the donor and target DNAs in vivo (14, 15). In contrast, prototypic Tn7 uses a cut-and-paste mechanism that directly forms a simple insertion (16) based on the heteromeric TnsA+TnsB transposase (17). TnsA and TnsB form a protein complex for which the nuclease activities of both proteins (TnsA and TnsB) are required to generate simple insertions (17–20), but the regulatory details of this process remain unresolved with Tn7 and related elements. A structure of the TnsB transposase would set the foundation for understanding the similarities that link related Tn7 and CAST elements, as well as the key differences that would explain their distinct behavior.

## Results

### TnsB and MuA Have Similar Architecture in the Context of the Strand-Transfer Complex.

**Fig. 1.**
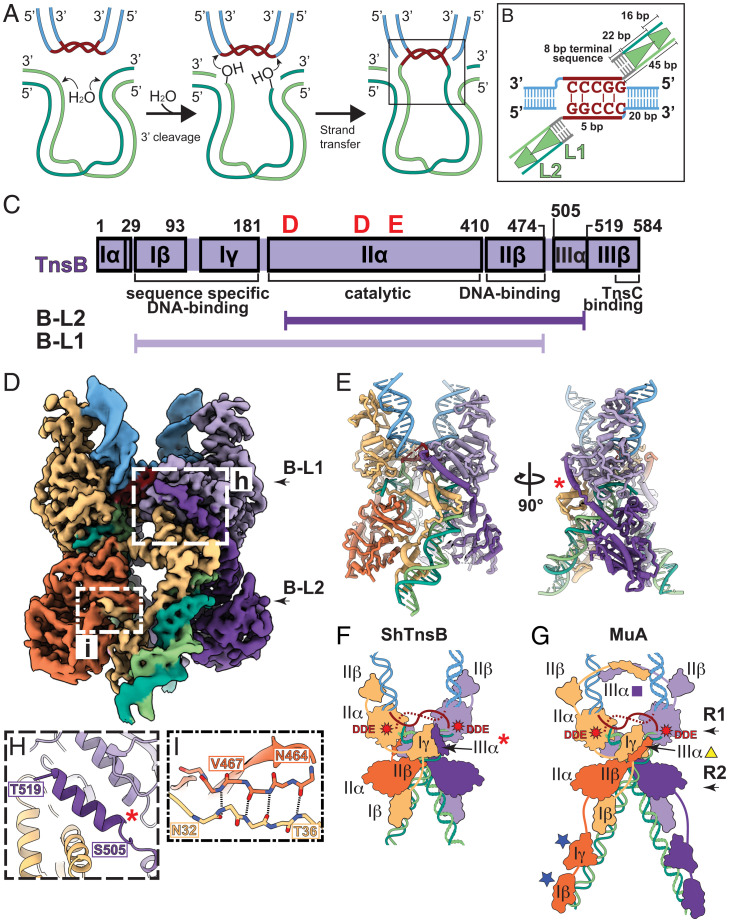
TnsB STC is a C2-symmetric tetramer with highly intertwined architecture. (*A*) Schematic of the TnsB transposition mechanism: transposon ends with flanking DNA (green), 5-bp target site (brown), and target DNA (blue). For simplicity, TnsB protomers are not shown. (*B*) Symmetric strand-transfer DNA substrate is designed to mimic the product of the strand-transfer reaction. The 45-bp part of the transposon left end (LE) includes 1.75 TnsB binding sites (one full binding site, corresponding to 22 bp of L1, and three-fourths of a binding site, corresponding to 16 bp of L2). TnsB binding sites overlap by 1 bp, indicated by green triangles. An 8-bp terminal sequence is colored gray. (*C*) Domain diagram of TnsB (purple; domain naming conventions follow MuA) and residue positions built for the two different conformations observed: B-L1 and B-L2 (explained further in *D*). (*D*) Cryo-EM reconstruction of the TnsB STC. TnsB subunits are referred to according to which TnsB repeat it binds at the transposon end, numbered starting from the target–donor junction. B-L1 is the TnsB monomer that binds the L1 TnsB DNA sequence, and B-L2 binds the L2 site. TnsB monomers are colored shades of purple or orange. B-L1 subunits (light purple and tan) and B-L2 subunits (dark purple and orange) form a C2-symmetric complex. DNA colors are identical with respect to *A*. (*E*) Atomic model of the TnsB STC. Two different views are shown; a red asterisk indicates the position of helix IIIα. (*F* and *G*) Architectural schematic of (*F*) the ShTnsB STC and (*G*) (PDB ID code 4FCY) the MuA STC from bacteriophage Mu. Helix IIIα is indicated with a red asterisk, yellow triangle, or purple square. The location of the DDE catalytic triad is indicated with red stars. Blue stars (DNA-binding domains) indicate domains that are present in MuA but not observed in the ShCAST TnsB STC structure. (*H* and *I*) Close-up view of intersubunit interactions in the TnsB STC. (*H*) Domain IIIα (indicated with a red asterisk) from B-L2 (residues 505 to 519) mediates a three-way interaction in between two B-L1 subunits. (*I*) B-L1 and B-L2 subunits form stabilizing beta-sheet interactions. Hydrogen bonds are indicated with dashed lines.

ShCAST transposition likely follows that of many other transposition systems: Pairing of the transposon ends (Fig. 1*A*, *Left*) is followed by nucleophilic attack at the transposon ends that allows them to be joined to target DNA (Fig. 1*A*, *Middle*), resulting in the product DNA, referred to here as the strand-transfer DNA (Fig. 1*A*, *Right*). To understand how TnsB recognizes and pairs the transposon ends and subsequently juxtaposes them to target DNA, we reconstituted and imaged a TnsB strand-transfer complex (STC) using a symmetric DNA substrate containing the first 45 bp of the ShCAST transposon’s left end (Fig. 1*B*) (4). This DNA sequence contains the first full TnsB binding site, L1, and three-fourths of the second TnsB binding site, L2, to mimic the product of transposition (Fig. 1*B*; see *SI Appendix* for details). Reconstitution with this substrate resulted in a homogeneous, stable assembly (as assessed by size-exclusion chromatography; *SI Appendix*, Fig. S1) with which we obtained a high-resolution cryo-electron microscopy (cryo-EM) reconstruction (3.7-Å global resolution; Fig. 1*D* and *SI Appendix*, Fig. S2).

Rigid-body docking of isolated domains obtained from an AlphaFold prediction (21) resulted in a nearly full-length atomic model spanning the majority of the TnsB sequence (GenBank accession no. WP_084763316.1; Fig. 1*C*). TnsB forms a C2-symmetric tetrameric assembly organized around the strand-transfer DNA (Fig. 1*D* and *E* and Movie S1). The overall architecture and arrangement of functional domains are remarkably similar to the MuA STC (22) (Fig. 1*F* and *G* and *SI Appendix*, Fig. S3). MuA is a well-studied RNaseH transposase that is responsible for bacteriophage Mu integration. In the representative view shown (Fig. 1*F* and *G*), both complexes resemble an “X,” where the upper half of the complex consists of the target DNA (blue, Fig. 1*F* and *G*) and the lower half consists of the transposon ends (green, Fig. 1*F* and *G*). Both MuA and TnsB cleave the donor DNA in *trans*—the subunit whose DNA-binding domain interacts with DNA on the right-hand side of the complex (tan subunit, Fig. 1*D* and *E*) positions the catalytic domain to interact with the DNA on the left side of the target–donor junction and vice versa (Fig. 1*D* and *E*). Furthermore, both left and right halves of the complexes are identical, with each half containing two protein chains, each in different conformations that are determined by where they bind on the DNA substrate. The two TnsB binding sites on the strand-transfer DNA substrate are referred to as L1 and L2 (because the designed DNA substrate used ShCAST left ends). The corresponding TnsB conformers are distinguished by which TnsB binding site they occupy (Movie S1), and hence the TnsB monomer bound to L1 is referred to as B-L1, and TnsB bound to L2 is referred to as B-L2 (Fig. 1*C* and *D*; both are described in more detail below).

We have assigned TnsB domain names following MuA domain names (Fig. 1*C*) (22), given the remarkable similarities between the STC structures, in order to facilitate structural comparisons. Domains Iβ, Iγ, and IIβ are DNA-binding domains (Fig. 1*C* and Movie S1), domain IIα is the catalytic domain, and, finally, domains IIIα and IIIβ span the TnsB C terminus, which will be discussed in detail in the following sections. The B-L1 conformation includes residues 29 to 474 and is positioned at the target–donor junction (tan and light purple, Fig. 1*C* and *D* and Movie S1). The second distinct conformation, B-L2, spans residues 196 to 519 (orange and dark purple, Fig. 1*C* and *D*) and binds the second TnsB binding site (L2).

### A Distinct Role for Helix IIIα in Stabilizing TnsB Strand-Transfer Architecture.

Compared with the MuA STC (22), two DNA-binding domains, Iβ and Iγ, from the TnsB B-L2 subunit are not present in our structure (compare Fig. 1*F* and *G*; domains present in MuA but absent in TnsB B-L2 are marked with a blue star in Fig. 1*G*), possibly due to the choice of substrate (our DNA contains an incomplete L2 TnsB binding site). We also observe structural differences between ShCAST TnsB and MuA assemblies. One example is how the tetrameric architecture is stabilized, most notably in the placement of helix IIIα (red asterisk, Fig. 1*F* and *G*). In MuA, helix IIIα adopts two different configurations in the R1- (purple square, Fig. 1*G*) and R2-bound MuA subunits (yellow triangle, Fig. 1*G*). In contrast, in the TnsB STC, helix IIIα appears to stabilize the tetramer by making different intersubunit interactions (Movie S1). B-L2 helix IIIα (red asterisk, Fig. 1*F*) wraps around the back of domain IIα of B-L1 (light purple, Fig. 1*E*) to nestle between the B-L1 (light purple and tan) subunits, forming interactions with both (Fig. 1*D* and *H*). In addition to helix IIIα, we observe intersubunit interactions between domain Iβ in B-L1 (tan, boxed in Fig. 1*I*) and domain IIβ in B-L2 (orange, Fig. 1*I*). Here, B-L1 domain Iβ completes a β-sheet within B-L2 domain IIβ (Fig. 1*I*). Therefore, while the TnsB STC contains many conserved features to ensure fidelity of synaptic complex assembly, it appears to have evolved different protein–protein interactions to hold the tetrameric assembly together compared with those found in the MuA STC.

### ShCAST Transposase Recruitment Occurs via Interactions between TnsC and TnsB’s C Terminus.

**Fig. 2.**
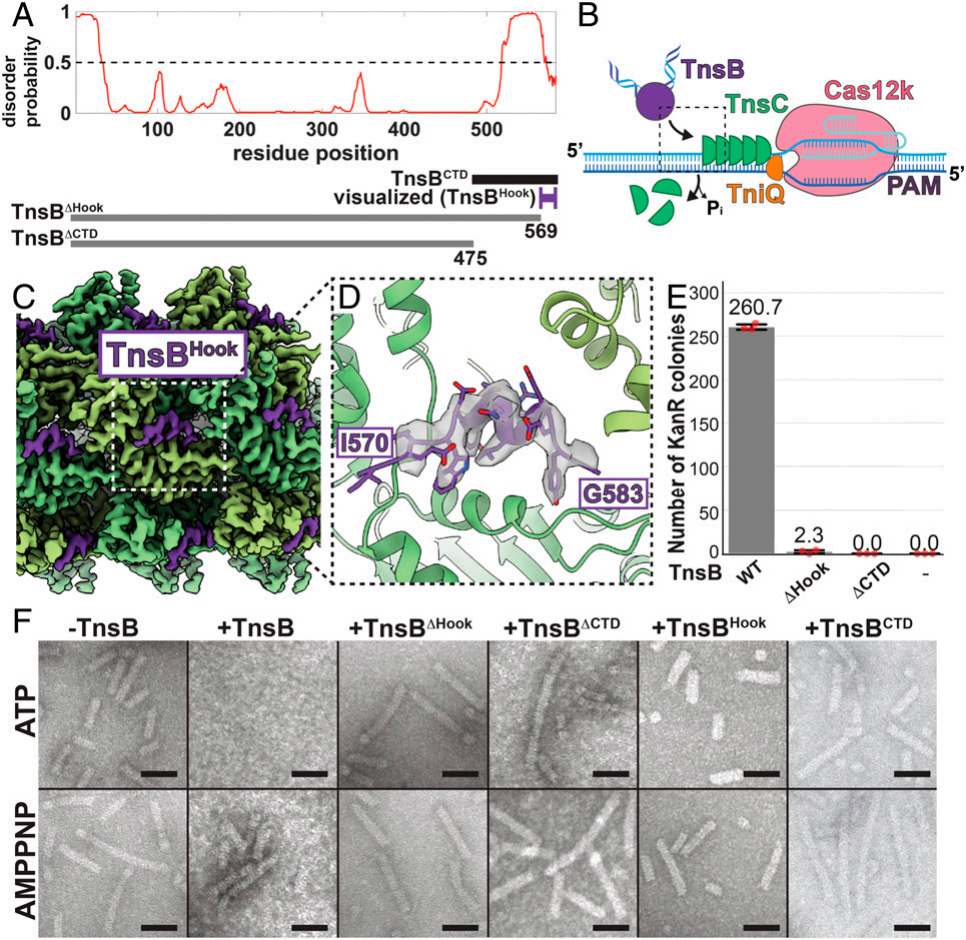
C terminus of TnsB decorates the TnsC filament, but TnsB binding is insufficient to stimulate TnsC filament disassembly. (*A*) Predicted disorder along the length of the TnsB sequence (full-length TnsB is 584 residues). Probabilities >0.5 (indicated with a dashed line) correspond to disordered prediction. The TnsB C-terminal domain (TnsB^CTD^, residues from 476 to 584; black bar) was used for cryo-EM structure determination, but only a fragment corresponding to TnsB^Hook^ (residues from 570 to 584; purple) was visualized in the cryo-EM reconstruction. Truncation of the hook (TnsB^ΔHook^; gray line) and C-terminal domain (TnsB^ΔCTD^; gray line) were designed for disassembly assays and in vitro transposition assays. (*B*) Schematic diagram of TnsB transposase (purple) recruitment (dashed black box) to the integration site defined by Cas12k (pink), TniQ (orange), and TnsC (green). (*C*) Cryo-EM reconstruction of the TnsC filament (green) coated with TnsB^Hook^ (purple). The close-up view in *D* is indicated with a white dashed box. (*D*) Cryo-EM density map of the TnsB^Hook^ fragment (transparent gray) with the TnsB^Hook^ atomic model (purple). Labeled amino acids indicate N- and C-terminal boundaries of the modeled portion of TnsB^Hook^ (the last residue at position 584 is not modeled). (*E*) Transposition activity observed with full-length TnsB (WT, wild type) is lost with C-terminal deletion constructs, TnsB^ΔHook^ or TnsB^ΔCTD^, comparable to the case in which no transposase is added (indicated with “-”). Transposition activity is assessed via the number of kanamycin-resistant (KanR) colonies (see *Materials and Methods* for details). Data are represented by the mean; error bars indicate SD (*n* = 3, technical triplicates). Raw data points are shown in red. (*F*) Negative-stain EM images indicate that TnsC filaments are disassembled in a nucleotide-dependent manner by full-length TnsB (compare AMPPNP and ATP), but not by any of the TnsB fragments tested. (Scale bars, 500 Å.)

We do not observe any ordered structure past domain IIIα in our TnsB STC structure (Fig. 1*C*), consistent with the disorder prediction in this region (Fig. 2*A*). Nevertheless, this is particularly interesting given the role of the transposase C terminus in both prototypic Tn7 and Mu. The last 22 residues of TnsB (residues 681 to 702) in prototypic Tn7 are essential for the TnsB–TnsC interaction and transposition (23). For Mu, the C terminus of MuA is crucial for stimulating adenosine triphosphate (ATP) hydrolysis (24) and disassembly of MuB filaments (the AAA+ protein providing a function analogous to ShCAST TnsC) (25, 26), implying that MuA C-terminal interactions with MuB are also relevant for MuA transposition. Motivated by the remarkable structural and functional similarities between MuB and ShCAST TnsC (12), we reasoned that the C-terminal 109 residues of TnsB (spanning domains IIIα and IIIβ, which we refer to as TnsB^CTD^; Fig. 2*A*) are most likely to interact with the TnsC filament. Because TnsB, like MuA, stimulates TnsC filament disassembly in a nucleotide-dependent manner (Fig. 2*B*) (12), we reasoned that full-length TnsB would not form a stable complex with TnsC filaments suitable for high-resolution structure determination, so we instead pursued structural characterization with TnsB fragments. In order to capture a homogeneous “recruitment-like” state, we added TnsB^CTD^ in excess to AMPPNP-bound TnsC, which forms continuous helical filaments on target DNA (12).

The cryo-EM reconstruction of the TnsC filament coated with TnsB^CTD^ revealed side-chain density features (3.5-Å resolution) corresponding to 14 residues decorating the surface of TnsC filaments (Fig. 2*C* and *SI Appendix*, Fig. S4). Atomic modeling into this density (*SI Appendix*, Fig. S5) indicated that this portion of TnsB most likely corresponds to the last 15 residues of TnsB (the last residue is not modeled), or residue positions 570 to 584, which we refer to as TnsB^Hook^ (Fig. 2*A* and *D*). Subsequent cryo-EM reconstruction of the TnsB^Hook^ peptide (residues 570 to 584; Fig. 2*A*) in the presence of TnsC filaments resulted in a reconstruction indistinguishable from the previous one, except for slight resolution differences (3.5 vs. 3.8 Å for the TnsB^CTD^ vs. TnsB^Hook^ reconstructions, respectively; *SI Appendix*, Fig. S6), confirming the TnsB^Hook^ sequence register assignment. The lack of additional density corresponding to TnsB^CTD^ in our cryo-EM reconstruction suggests positions outside the structured TnsB^Hook^ do not make specific contacts with the TnsC filament, which is consistent with TnsB disorder predictions (Fig. 2*A*). Taken together, the most parsimonious explanation for this is that the TnsB^Hook^ represents a structured interaction with TnsC connected by a flexible linker to the rest of the full-length TnsB. Deletions of either the TnsB^CTD^ (ΔCTD, or equivalently TnsB^ΔCTD^, corresponding to residues 1 to 475; Fig. 2*A*) or the TnsB^Hook^ (ΔHook, or TnsB^ΔHook^, corresponding to residues 1 to 569; Fig. 2*A*) result in loss of transposition activity (Fig. 2*E*).

ShCAST target-site selection relies on the stimulation of TnsC filament disassembly by TnsB-promoted ATP hydrolysis to allow guide RNA–directed transposition (Fig. 2*B*) (12). Therefore, we wondered whether interactions with the TnsC filament were sufficient for hydrolyzable nucleotide-dependent filament disassembly, as observed in Mu (24, 27). However, none of the TnsB N-terminal (TnsB^ΔHook^: 1 to 569; TnsB^ΔCTD^: 1 to 475) or C-terminal fragments (TnsB^Hook^: 570 to 584; TnsB^CTD^: 476 to 584) we assayed were sufficient to recapitulate the disassembly phenotype observed with full-length TnsB with ATP using EM imaging (Fig. 2*F*) or biochemical assays that track TnsC oligomerization on DNA (*SI Appendix*, Fig. S7), at least at concentrations for which full-length TnsB is effective at stimulating TnsC filament disassembly. Therefore, in contrast to Mu (24), this suggests that one or more additional interactions between TnsB and TnsC, in addition to that made with the TnsB^Hook^, are required in order to stimulate ATP hydrolysis and filament disassembly in ShCAST. Although a MuA–MuB structure is not available, the interaction surface between TnsB and TnsC appears to colocalize to the same interaction surface mapped to MuB, assuming positions between TnsC and MuB are roughly equivalent (*SI Appendix*, Fig. S8*A*) (27, 28). Nevertheless, the lysine residues responsible for mediating transposase interactions in MuB do not appear conserved (*SI Appendix*, Fig. S8*B*), suggesting that the nature of interactions between the transposase and its AAA+ regulator varies across transposition systems.

Together, these results paint a picture of the initial steps of TnsB recruitment to the target site via the AAA+ regulator, TnsC. TnsB’s C-terminal hook interacts with TnsC along the surface of the filament, but interaction via the TnsB^Hook^ alone is insufficient to stimulate TnsC filament disassembly, indicating that one or more additional interactions between TnsB and TnsC not visualized here must be required. In addition, we reveal that the TnsB C-terminal hook is flexibly linked to the rest of TnsB. The flexible linker is not conserved in length or sequence among TnsB homologs from the V-K CAST elements (*SI Appendix*, Fig. S9). Nevertheless, given the relatively precise insertion spacing observed in ShCAST (4), it may play crucial roles in orienting TnsB to interact productively with the target site.

### The TnsB Strand-Transfer Complex Stabilizes Highly Distorted DNA.

**Fig. 3.**
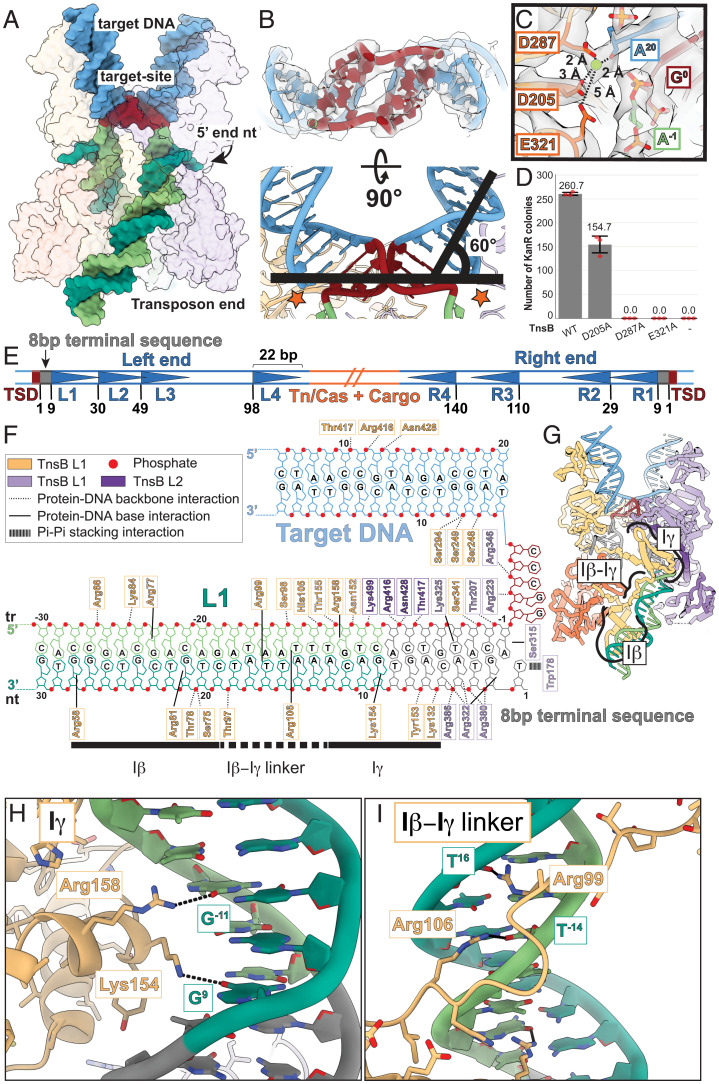
Target-DNA distortions and transposon end-binding sequence requirements are revealed in the TnsB STC. (*A*) Atomic model of the strand-transfer DNA. Protein is shown with a transparent surface. Target DNA colored is blue; the target site is colored brown. Transposon end DNA is colored in different shades of green: The transferred strand (tr) is colored light green; the nontransferred strand (nt) is colored dark green. (*B*) Target DNA (brown) is highly distorted, bent at each end by 60°. Stars indicate the target–donor junction. (*C*) DDE catalytic residues (orange) are displayed along with distances to the Mg^2+^ ion (dashed line), close to the scissile phosphate on the strand-transfer substrate. Cryo-EM map density is shown in transparent surface. (*D*) Alanine mutation of the catalytic residues (D205A, D287A, and E321A) results in significant loss of transposition activity. Negative control without TnsB is indicated as “-.” Transposition activity was assessed by the number of KanR transformants. Data are represented by the mean; error bars indicate SD (*n* = 3, technical triplicates). Raw data points are shown in red. (*E*) The structure of ShCAST transposon ends following integration. The target-site duplication (TSD; brown) is adjacent to an 8-bp terminal sequence (gray) and flanked by multiple TnsB binding sites (blue triangles). Flanking host target DNA is shown in light blue, and transposon/CRISPR–associated (Tn/Cas) genes and cargo DNA are in orange. ShCAST transposon ends are characterized by four unevenly and nonsymmetrically spaced TnsB binding sites (represented as blue triangles, L1 to L4 and R1 to R4). Base pair positions (numbered) indicate the start of each TnsB binding site. (*F*) Diagram of protein–DNA interactions made within the STC core, along the 8-bp terminal sequence, and the first TnsB binding site. Both base-specific contacts (solid line) and interactions with the sugar-phosphate backbone (dashed line) are made throughout the terminal inverted repeat as well as with the target DNA. Notable pi–pi stacking arrangements (indicated with a wide dashed line) are also shown, which interact with the 5′ end of the nontransferred strand. Domains of TnsB that interact with the L1 TnsB-binding sequence are marked. (*G*) Specific domains in TnsB (Iβ, Iγ, and Iβ–Iγ linker) are indicated in the model overview, which are shown as close-up insets in *H* and *I*. (*H* and *I*) Base-specific contacts are made throughout the TnsB binding site with the TnsB Iγ domain (*H*) and TnsB Iβ–Iγ linker (*I*), highlighting the mechanistic basis of transposon end recognition by TnsB. Possible hydrogen-bonding interactions are indicated with dashed black lines.

DNA distortions, particularly in the target-bound DNA, are canonical features of RNaseH transposase structures. The TnsB STC has highly distorted DNA (120° bend; Fig. 3*A* and *B*) surrounding the 5-bp target site (brown, Fig. 3*A*) comparable to MuA (22). Target DNA distortions are required to place the scissile phosphate appropriately in the active site (29). Consistent with this, the DDE catalytic residues (D205, D287, and E321; Fig. 3*C*) are positioned at the target–donor junction precisely at the DNA distortion (red star, Fig. 3*B*), coordinating a magnesium ion with the scissile phosphate poised for nucleophilic attack (Fig. 3*C*). Mutation of the catalytic residues significantly reduced transposition activity (Fig. 3*D*). Surprisingly, the D205 mutation did not completely abolish transposition, but there is no immediately nearby acidic residue that can compensate for the role of D205 (the closest Asp/Glu residue is D291, which is 7.2 Å away from the Mg^2+^ ion). Thus, it requires further investigation to understand how the D205A mutant can still carry out transposition.

In MuA, helix IIIα of the R1-bound subunit (light purple, indicated with a purple square, Fig. 1*G*) has additional roles in stabilizing target-DNA distortions and preventing reversal of the reaction (30). The absence of a similar interaction in the TnsB STC structure (Fig. 1*F*) suggests that the role of helix IIIα in TnsB may primarily be for tetramer stabilization rather than stabilizing target-DNA distortions. Consistent with this, in TnsB the domain IIβ close to the target DNA is closely interacting with the sugar-phosphate backbone, whereas the equivalent domain in MuA is too far to interact with the target DNA (*SI Appendix*, Fig. S10). This suggests that target-DNA distortions in TnsB are stabilized via a different DNA-binding domain, namely domain IIβ.

### TnsB Interactions with Donor DNA Delineates Transposon End Recognition.

Tn7-like elements have an 8-bp terminal sequence (gray, adjacent to the target-site duplication, and target site in brown, Fig. 3*E*) (2). In our structure, the 8-bp terminal sequence (closest to the target-site duplication and colored gray, Fig. 3*E*) corresponds to the part of the DNA substrate contacted by the catalytic domain (domain IIα; *SI Appendix*, Fig. S11) and can be assigned to the contacts between the B-L1 subunit and target DNA near the target–donor junction (Fig. 3*F*). Transposon cargo and Tn7/CRISPR–associated genes are flanked by left and right ends, consisting of multiple 22-bp TnsB binding sites (1, 2, 31) (blue triangles, Fig. 3*E*). In order to understand the protein–DNA interactions that enable TnsB to recognize its cognate DNA sequence, we looked at DNA-binding domains Iβ and Iγ which bind along the first TnsB binding site on the donor DNA (L1; Fig. 3*F* and *G*). The majority of protein–DNA interactions are sequence-nonspecific contacts with the sugar-phosphate backbone (Fig. 3*F*). However, several key residues located in the Iγ domain and in the Iβ–Iγ linker form sequence-specific nucleobase contacts. Within Iγ, R158 and K154 are within hydrogen-bonding distance of G^−11^ and G^9^, respectively (Fig. 3*H*). Interestingly, the Iβ–Iγ linker lies along the minor groove of the DNA duplex and contributes sequence-specific contacts. R106 and R99 are within hydrogen-bonding distance of T^−14^ and T^16^, respectively (Fig. 3*I*). The Iγ and Iβ–Iγ linker makes contact with nucleotide positions 5 to 19, which is roughly consistent with the pattern of conservation among TnsB binding sites (*SI Appendix*, Fig. S12). Although some base-specific interactions are observed in the Iβ domain (R58, R77, and R81), the lack of strong conservation in the TnsB donor sequence in this region (positions 20 to 30; *SI Appendix*, Fig. S12) suggests that these residues may not strongly contribute to transposon end recognition. Therefore, the TnsB STC structure suggests that transposon end DNA recognition may be modular (i.e., independent and separable from catalytic function) in TnsB, like MuA (32), and could feasibly be altered using rational design strategies, as has been done in the past with MuA via the generation of a chimeric recombinase called “SinMu” (33).

### TnsB Forms Specific Base-Stabilizing Contacts in the Nontransferred Strand.

**Fig. 4.**
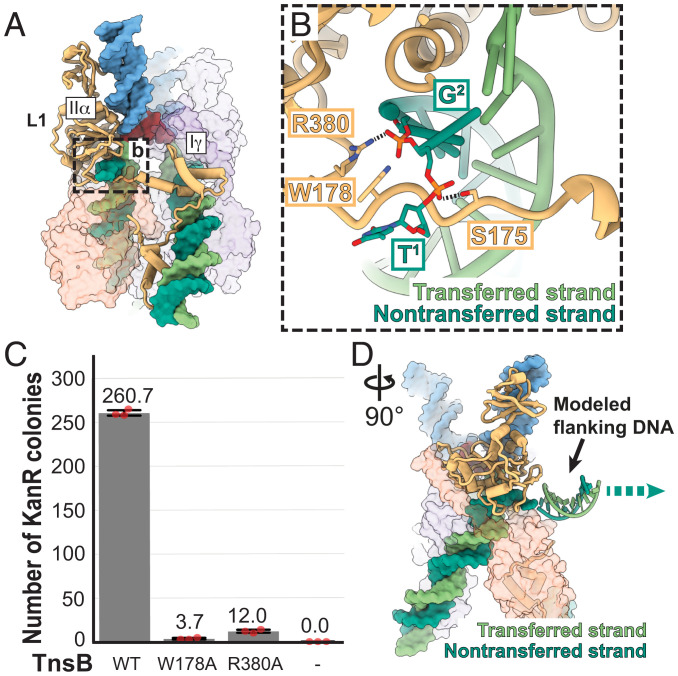
Base flipping at the 5′ transposon end is stabilized by TnsB, which is important for transposition. (*A*) The 5′ end of the element at the nontransferred strand (dark green) is stabilized by a flexible linker connecting domain IIα and domain Iγ of the B-L1 subunit (tan). (*B*) The 5′ end of the element at the nontransferred strand is melted and the first position (T^1^) is flipped out. Recognition of the nontransferred strand is mediated by hydrogen-bonding (dashed black lines) and pi–pi stacking interactions by the labeled residues. (*C*) Mutation of the residues shown to stabilize the 5′ end of the nontransferred strand results in near-complete loss of transposition activity. The number of KanR colonies was used as a proxy for the transposition activity from the in vitro transposition assay. Data are represented by the mean; error bars indicate SD (*n* = 3, technical triplicates). Raw data points are shown in red. (*D*) Modeled flanking DNA reveals that noncleaved double-stranded flanking DNA is compatible with the TnsB STC structure reported here. The direction of flanking DNA is indicated with an arrow.

Unlike prototypic Tn7 or other CAST elements (such as the I-F3 subfamily), ShCAST (and other V-K CAST elements) do not encode enzymatic activity for cleavage at the 5′ ends of the element (i.e., it does not encode TnsA) (15). Consistent with this, CAST V-K elements form cointegrates indicative of replicative transposition without subsequent resolution (14). Therefore, we were particularly intrigued to discover a unique structural conformation at the 5′ ends of the transposon (and missing in MuA) with the nontransferred strand (Fig. 4*A*). The linker connecting domains Iγ and IIα in the L1-bound TnsB subunit snakes underneath each 5′ end of the element in the nontransferred strand (Fig. 4*A*), forming stabilizing interactions (Fig. 4*B*) with the first two nucleotide positions. We observe “melting” of the 5′ end of the nontransferred strand through a flipped-out base (T^1^; Fig. 4*B*). This specific conformation is stabilized by aromatic interactions with W178 and hydrogen bonding with S175 and R380 (Fig. 4*B*). Mutation of residues observed to interact with the nontransferred strand results in almost complete abrogation of transposition activity (Fig. 4*C*), highlighting the importance of the observed interactions.

We wondered whether specific interactions at the ends of the element were consistent with additional flanking DNA from the donor plasmid, as would be expected given TnsB’s transposition mechanism (Fig. 1*A*). Modeled flanking DNA from the 5′ end of the transposon is sterically accommodated within our existing structure (Fig. 4*D*), indicating that the DNA substrate we used here is consistent with formation of TnsB cointegrates. Therefore, it appears that the specific structural feature we observe at the 5′ end of the element is both important and consistent with TnsB’s expected transposition substrate. We postulate that the melting of the 5′ nontransferred strand may serve as a regulatory step that ensures the fidelity of synaptic complex assembly.

## Discussion

The structures reported here include an STC of a Tn7-like CAST element, and also highlight the remarkable consistency across the catalytic domains of RNaseH transposases, specifically with respect to the Mu transposase (22), despite distant evolutionary relationships. This high degree of structural conservation across considerable evolutionary distance leads us to propose that TnsB from prototypic Tn7 adopts an architecture similar to ShCAST TnsB and MuA upon integration. While not addressed in this work, multiple internal TnsB binding sites found asymmetrically in the left and right ends (Fig. 3*E*) must somehow establish the strict orientation specificity found with these elements (3, 4, 34). Therefore, a lingering mystery for ShCAST and related transposons is how placement of internal binding sites establishes the orientation and fidelity of synaptic complex assembly.

AlphaFold predictions of the catalytic domain (domain IIα) of prototypic Tn7 TnsB superimposes well onto ShCAST TnsB (2.4 Å rmsd; *SI Appendix*, Fig. S13*A*). Interestingly, the region known to interact with TnsA in the prototypic Tn7 system (19) localizes to where flanking host DNA would be located (*SI Appendix*, Fig. S13*B*). Given this is the position where the TnsA nuclease would need to localize in order to generate 5′ end cuts for generating simple insertions, this structure suggests that manipulation of ShCAST transposon characteristics via structure-based engineering is practically achievable.

The structural features we observe at the 5′ transposon end in the STC structure (Fig. 4*B*) have also been similarly observed in the RAG1–RAG2 synaptic complex in which a base-flipping mechanism is important for end recognition and stabilization of the heptameric RSS sequence (35). In contrast, analogous base flipping is not observed in the MuA structure (22), which is not completely modeled in this region. The absence of this feature in MuA is either a result of lack of resolution (due to anisotropic resolution) or, alternatively, that Mu does not stabilize nicked ends in an identical manner compared with ShCAST TnsB. Further research will be required to understand the exact functional role for base flipping in these elements.

The structural work described here also sheds light onto the process of transposase recruitment to the target site for ShCAST and related transposition systems. We demonstrate that physical association between TnsB^CTD^ and TnsC is primarily via the C-terminal hook that is capable of decorating TnsC filaments (Fig. 2). A total of 50 residues (520 to 569) are not observed in either our TnsB^CTD^–TnsC structure nor the TnsB STC structure, and are consistent with predictions of disorder based on primary sequence (Fig. 2*A*). This suggests that this particular region of TnsB remains flexible and without structure, at least in the states we have captured here. This is consistent with a model in which a second interaction between TnsB and TnsC is required to recapitulate nucleotide-dependent TnsC filament disassembly, which is observed with full-length TnsB but not the TnsB fragments that we used to decorate TnsC. Such interactions may also be needed to activate the otherwise latent transposition activity in ShCAST TnsB. While the structures here reveal mechanistic insight into TnsB function and provide a basis for ShCAST engineering, this work also uncovers exciting questions centered on ShCAST transposon structure and function that will remain fascinating topics for future investigations.

## Materials and Methods

### Strand-Transfer Complex Reconstitution.

The strand-transfer DNA substrate was prepared by annealing three oligonucleotides, heating to 95 °C, and then cooling slowly to room temperature in annealing buffer (*SI Appendix* for composition) (*SI Appendix*, Table S2). The strand-transfer DNA substrate and purified TnsB were mixed in a 1:6 molar ratio with the following final buffer composition: 26 mM Hepes (pH 7.5), 5 mM Tris⋅HCl (pH 7.5), 20 mM KCl, 100 mM NaCl, 0.2 mM MgCl_2_, 15 mM MgOAc_2_, 3% glycerol, and 1.5 mM dithiothreitol (DTT). After incubation at 37 °C for 40 min, the sample was concentrated to ∼7 mg/mL using an Amicon Ultra centrifugal filter (50-kDa molecular weight cutoff, EMD Millipore); 250 μL of the concentrated sample was subjected to size-exclusion chromatography (Superdex S200 Increase 10/300 GL, Cytiva). Peak fractions from 9.2 to 10.7 mL were collected for cryo-EM sample preparation (*SI Appendix*, Fig. S1).

### TnsB^CTD^–TnsC Complex Preparation.

TnsB and TnsC were purified following previously described protocols (4, 12). Protein truncation constructs consisting of TnsB’s 109 C-terminal residues (referred to throughout as TnsB^CTD^) were cloned from the ShTnsB vector (Addgene, 135525) and purified using previously described protocols (4, 12). To prepare the TnsB^CTD^–TnsC complex for cryo-EM imaging, TnsC filaments were formed by mixing purified TnsC with a 1:10 molar ratio of a 22-bp double-stranded DNA (dsDNA) substrate (*SI Appendix*, Table S2; see *SI Appendix* for more details). TnsC was allowed to polymerize on ice for 5 min before adding purified TnsB^CTD^ at a twofold molar excess with respect to TnsC.

### Cryo-EM Sample Preparation and Imaging.

Slightly different sample preparation protocols were used for the two samples (referred to as TnsB STC and TnsB^CTD^–TnsC) described in this manuscript. For the TnsB STC, homemade graphene oxide (GO) grids were used (*SI Appendix* for fabrication details); 4 μL of reconstituted TnsB STC was loaded onto the carbon side of freshly fabricated GO grids. The sample was incubated on the grid for 20 s in the chamber of a Mark IV Vitrobot (ThermoFisher), which was set to 4 °C and 100% humidity. Grids were blotted using a blot force of 5 and blot time of 7 s prior to being plunged into liquid ethane cooled by liquid nitrogen. For the TnsB^CTD^–TnsC, R1.2/1.3 gold grids (UltraAuFoil, Quantifoil) were glow-discharged (PELCO easiGlow) using a 30-mA current for 30 s prior to sample application and vitrification; 4 μL of freshly reconstituted TnsB^CTD^–TnsC sample was applied to the gold grid. Vitrification conditions followed that of the TnsB STC (see above).

Vitrified samples were imaged using a Talos Arctica (ThermoFisher, operated at 200 keV) equipped with a BioQuantum energy filter (Gatan) and a K3 direct electron detector (Gatan). The microscope was subjected to stringent alignment procedures, including coma-free alignment and parallel illumination (36). High-throughput imaging was achieved using a 3-by-3 image shift in SerialEM (37). Image magnification settings corresponded to 63,000× magnification (1.33 Å per pixel scaling) and a nominal defocus range of −1.0 to −2.5 μm. Comprehensive imaging parameter details are presented in *SI Appendix*, Table S1.

### Image Processing.

Warp (38) was used for micrograph preprocessing, including beam-induced motion correction, contrast transfer function (CTF) estimation, and initial particle picking. For the TnsB STC, a C1 ab-initio reconstruction was generated using cryoSPARC (39). At this point, the resulting reconstruction had apparent C2 symmetry, therefore C2 symmetry was imposed for all subsequent refinement steps. For the TnsB^CTD^–TnsC reconstruction, a 20-Å low pass–filtered map of the ATPγS-bound TnsC cryo-EM reconstruction (EMD-23720) (12) was used as an initial reference for cryoSPARC helical reconstruction and refinement (39). Roughly the same refinement procedure was applied to both datasets: cryoSPARC particle alignment parameters and stacks were exported to RELION (40, 41) for subsequent refinement, including three-dimensional classification, CTF refinement (42), and Bayesian polishing (43). The final TnsB STC reconstruction had an estimated resolution of 3.7 Å (gold standard Fourier shell correlation, [FSC]) and, in the case of the TnsB^CTD^–TnsC reconstruction, 3.5-Å resolution. More comprehensive methodological details are presented in *SI Appendix*.

### Atomic Model Building.

Different modeling procedures were used for the TnsB STC and TnsB^CTD^–TnsC filament cryo-EM reconstructions. For the TnsB STC cryo-EM map, the TnsB sequence was used to generate an AlphaFold2 (21) prediction. The top-ranked model was split by domain and manually docked into the cryo-EM map using UCSF Chimera (44). One half of the complex, containing two distinct conformations of TnsB and DNA, was completed manually using Coot (45) and C2 symmetry was used to generate the full complex. This was followed by manual inspection and further refinement using Coot (45). The full assembly was energy-minimized in the context of the cryo-EM map using Rosetta (46). Protein and DNA geometry was subjected to Phenix real-space refinement (47).

In the case of the TnsB^CTD^–TnsC filament cryo-EM reconstruction, TnsC and DNA models from the ATPγS-bound TnsC filament (Protein Data Bank [PDB] ID code 7M99) served as very close initial models. Small adjustments in the TnsC model were made using Rosetta energy minimization, employing helical symmetry to model two helical turns using a single asymmetric unit, as described previously (12). In order to identify the register of the TnsB^Hook^ fragment, a 14-residue polyalanine backbone was first built into the density. A custom script was used to thread all 96 possible registers, representing each possible threaded sequence spanning the 109-residue TnsB^CTD^ construct (109 − 14 + 1 = 96 possible registers), onto the TnsB fragment backbone. Each initial model was then relaxed into the density independently, using Rosetta energy minimization (46). Additional Rosetta energy terms to assess atomic model-map fit (elec_dens_fast weight = 40) were enforced during refinement (*SI Appendix*, Fig. S5), and 30 models were generated for each energy minimization run. The best scoring model was used to assess the sequence register, as shown in *SI Appendix*, Fig. S5. Details of the model statistics and validation are presented in *SI Appendix*, Table S1.

### In Vitro Transposition Assay.

In vitro transposition assays were carried out as previously described (4, 12). First, 48 μL of the target pot reaction (containing pTarget_PSP1 Addgene 127926, Cas12k, single-guide RNA [sgRNA], TnsC, and TniQ) and 48 μL of the donor pot reaction (containing pDonor_ShCAST Addgene 127924 and TnsB) were independently incubated at 37 °C for 15 min. Then, these two pots were combined and supplemented with 4 μL of 375 mM MgOAc_2_. The mixture was then incubated for 2 additional hours at 37 °C. The combined final transposition reaction consisted of the following: 50 nM Cas12k, 50 nM TnsC, 50 nM TniQ, 50 nM TnsB, 100 nM sgRNA, 26 mM Hepes (pH 7.5), 4 mM Tris (pH 7.4), 40 mM NaCl, 10 mM KCl, 0.8% glycerol, 2 mM DTT, 50 μg/mL bovine serum albumin, 0.04 mM ethylenediaminetetraacetate, 0.2 mM MgCl_2_, 15 mM MgOAc_2_, 0.54 nM pDonor_ShCAST, 0.45 nM pTarget_PSP1, and 2 mM ATP. Product DNA was purified from the reaction mixture using a GeneJET PCR Purification Kit (ThermoFisher), followed by heat-shock transformation into DH5α competent cells (a gift from the J.E.P. laboratory). Transformed cells were plated on agar plates with 50 μg/mL kanamycin for selection.

### TnsC Filament Disassembly Assay.

TnsC disassembly was probed using two different assays: an EM-based imaging assay and a biochemical assay. The imaging assay was carried out as follows: TnsC and 60-bp dsDNA (*SI Appendix*, Table S2) were added at a 25:1 molar ratio into the following buffer: 2 mM nucleotide (ATP or AMPPNP), 25 mM Hepes, 200 mM NaCl, 2% glycerol, 1 mM DTT, and 2 mM MgCl_2_ in order to initiate filament assembly. Filaments were then either incubated with full-length TnsB or TnsB truncations (1:1 molar ratio of TnsC to TnsB) or an equivalent volume of the buffer as a negative control. Each reaction was incubated at 30 °C for 1 h, followed by negative-stain EM. For the biochemical assay, desthiobiotinylated DNA was incubated with streptavidin magnetic beads. TnsC filament assembly was initiated (as described above) and then TnsB (full-length or truncation constructs) was added to the reaction mixture. After multiple washes, DNA was eluted from the beads using a 4 mM biotin solution and the associated proteins were examined using sodium dodecyl sulfate–polyacrylamide gel electrophoresis. *SI Appendix* includes more comprehensive details.

## Supplementary Material

Supplementary File

Supplementary File

## Data Availability

Electron density maps and atomic models reported in this article have been deposited in the Protein Data Bank and Electron Microscopy Data Bank (EMDB) (EMDB ID code 25454 TnsB^CTD^-TnsC filament, EMDB ID code 25455 TnsB STC, EMDB ID code 27140 TnsB^Hook^-TnsC filament, PDB ID code 7SVV TnsB^CTD^-TnsC filament, and PDB ID code 7SVW TnsB STC). All study data are included in the article and/or supporting information.
