# Supplementary material for: Mechanistic details of CRISPR-associated transposon recruitment and integration revealed by cryo-EM
Source: Proc Natl Acad Sci U S A. 2022 Aug 1;119(32):e2202590119. doi: 10.1073/pnas.2202590119 (PMC9371665; doi:10.1073/pnas.2202590119)
Supplement: Supplementary File [file pnas.2202590119.sapp.pdf]

Extended Materials and Methods

TnsB sequence information

The full-length TnsB sequence used throughout is below:

> **TnsB, GenbankID WP_084763316.1**

MNSQQNPDLAVHPLAIPMEGLLGESATTLEKNVIATQLSEEAQVKLEVIQSLLEPCDRTTYGQK
LREAAEKLNVSLRTVQRLVKNWEQDGLVGLTQTSRADKGKHRIGEFWENFITKTYKEGNKGSK
RMTPKQVALRVEAKARELKDSKPPNYKTVLRVLAPILEKQQKAKSIRSPGWRGTTLSVKTREG
KDLSVDYSNHVWQCDHTRVDVLLVDQHGEILSRPWLTTVIDTYSRCIMGINLGFDAPSSGVVAL
ALRHAILPKRYGSEYKLHCEWGTYGKPEHFYTDGGKDFRSNHLSQIGAQLGFVCHLRDRPSE
GGVVERPFKTLNDQLFSTLPGYTGSNVQERPEDAEKDARLTLRELEQLLVRYIVDRYNQSIDAR
MGDQTRFERWEAGLPTVPVPIPERDLDICLMKQSRRTVQRGGCLQFQNLMYRGEYLAGYAGE
TVNLRFDPRDITTILVYRQENNQEVFLTRAHAQGLETEQLALDEAEAASRRLRTAGKTISNQSLL
QEVVDRDALVATKKSRKERQKLEQTVLRSAAVDESNRESLPSQIVEPDEVESTETVHSQYEDIE
VWDYEQLREEYGF

Strand-transfer product DNA

The strand-transfer product DNA substrate (stDNA) was designed to mimick the product DNA, following the strategy used to stabilize the P-element transpososome (1). This strategy relies on the generation of a symmetric, branched DNA substrate that mimicks the product of integration. Because the target-site duplication is an odd number of basepairs (5 bp), the product substrate unavoidably contains a single mis-match in the middle of the target-site. Based on the sequence similarity between TnsB transposon right and left ends for the first two TnsB binding sites (L1, L2, R1, and R2, Figure S12), we suspect that substrate designs based on TnsB right transposon ends would have yielded similar results. Three oligonucleotides were used to reconstitute stDNA: LE_STC_For, LE_STC_Rev1, and LE_STC_Rev2 (Table S2). A 100 μM stock of three oligonucleotides were mixed in 1:1:1 molar ratio to make 30 μM final concentration of stDNA. 10X annealing buffer was added to the solution to result in the following buffer composition: 10 mM Tris pH 7.5, 50 mM NaCl, and 1 mM EDTA. The mixture was heated up to 95°C for 10 min and slowly cooled down to 4°C.

Strand-transfer complex reconstitution

TnsB was purified as previously described (2, 3). Purified TnsB was concentrated to 4.5 mg/mL, flash-frozen using liquid nitrogen, and stored at -80°C. TnsB was defrosted immediately before use and centrifuged at 21,000 rcf for 10 min to remove possible aggregation. stDNA (described above) and TnsB were mixed in 1:6 molar ratio with the following final buffer composition: 26 mM HEPES pH 7.5, 5 mM Tris-HCl pH 7.5, 20 mM KCl, 100 mM NaCl, 0.2 mM $MgCl_2$, 15 mM $MgOAc_2$, 3% Glycerol, and 1.5 mM DTT. Reconstituted STC was incubated at 37°C for 40 min and concentrated to ~7 mg/mL using an Amicon Ultra Centrifugal filter (50 kDa MWCO, EMD Millipore). 250 μL of the concentrated sample was subjected to size-exclusion chromatography (Akta Pure, Cytiva) using a Superdex 200 Increase 10/300 GL sizing column (Cytiva). The peak corresponding to fractions 9.2 mL to 10.7 mL were consolidated and concentrated to ~1 mg/mL using an Amicon Ultra Centrifugal filter (50 kDa MWCO, EMD Millipore). As a control, strand-transfer product DNA and TnsB protein were independently subjected to the size-exclusion chromatography using identical conditions (Figure S1).

Graphene oxide grid preparation

Graphene oxide (GO) was synthesized using previously published protocols (4, 5). Synthesized GO was deposited onto C-Flat R1.2/1.3 Au 300 mesh grids (Electron Microscopy Sciences) using a slightly modified deposition procedure (6, 7). Grids were individually prepared in batches. We describe the approach as follows: grids were individually washed with droplets of chloroform, followed by 1 minute glow discharging (easiGlow PELCO) set to 0.38 mBar and 15 mA conditions. 4 μL of 1 mg/mL PEI (diluted into 22.5 mM HEPES pH 7.5) was added to the carbon side of a grid, incubated for 2 minutes, and then the remaining PEI solution was blotted off using filter paper (Whatman). The carbon side of the grid was immediately washed twice in 20 μL ddH_2O droplets

and blotted using filter paper (Whatman). The grids dried for 5 minutes before 3 μL of 0.04 mg/mL graphene oxide solution was applied to the carbon side of each grid. The solution was incubated on the grid for 1 minute, followed by blotting and immediate washing with three 20 μL ddH_2O droplets (in the same fashion as described above). The remaining solution was blotted from each grid using filter paper (Whatman). Batches of prepared grids were dried for 10 minutes prior to sample application and vitrification.

TnsB Strand-transfer Complex (STC) sample preparation and cryo-EM imaging

TnsB-STC samples were prepared by diluting reconstituted STC samples to 0.4 mg/mL. 4 μL of STC sample was loaded on the carbon-side of a graphene oxide coated grid and incubated for 20 seconds in the vitrobot chamber (ThermoFisher), which was set to 4 °C and 100% humidity. Each grid was blotted for 7 seconds with blot force 5, and then plunged into liquid ethane cooled with liquid nitrogen. Vitrified grids were imaged using Talos Arctica (ThermoFisher, 200 keV) equipped with K3 direct electron detector (Gatan) and BioQuantum energy filter (Gatan). The microscope was aligned carefully prior to the data collection using established procedures for parallel illumination and coma-free alignments (8). 1,925 micrographs were collected at the nominal magnification of 63,000X (corresponding to 1.33 Å per pixel) using SerialEM (9), with 3 by 3 image shift and nominal defocus with range: -1.0 μm to -2.5 μm. Movies were fractionated into 49 frames, corresponding to a total dose of 49 electrons / $Å^2$ spread over 3 seconds.

TnsB STC Image processing

Warp (10) was used for image pre-processing, consisting of beam-induced motion correction, CTF estimation, and initial particle picking. A subset of the particle stack from Warp BoxNet was used to train Topaz (11) neural network, which yielded 186,121 particles. These particles were imported to cryoSPARC (12) for initial model generation. 2D classification revealed C2 symmetric

particles. Rejection of poorly resolved 2D classes resulted in 75,627 particles. *Ab initio* model generation using cryoSPARC (without imposing symmetry) resulted in a suitable initial model which was used for downstream refinement. After non-uniform refinement in cryoSPARC, the reconstruction and particle stack were imported to RELION (13) for subsequent image processing. RELION 3D classification (skipping alignments) was used to further improve structural homogeneity, which gave us the final particle stack (46,568 particles). Iteration of CTF refinement (14), Bayesian polishing (15), and 3D refinement in RELION improved the reconstruction to the final reconstruction shown, 3.69 Å.

STC Atomic Model Building

An initial atomic model representing the STC cryo-EM reconstruction was generated using AlphaFold2 (16). The top AlphaFold2 model produced using the TnsB sequence was split by domain and manually docked into the cryo-EM density using UCSF Chimera (17). Coot (18) was used to build the loops between domains, and refine the structure into the cryo-EM density. Each subunit of B-L1 and B-L2 was independently built and relaxed into the density using Rosetta energy minimization (19). C2 symmetry was applied to each conformer in order to generate the full assembly. DNA and Magnesium ions were manually fitted into the cryo-EM density using Coot (18). The modeled DNA substrate was subjected to real-space refinement using phenix_real_space_refine (20, 21), with manually defined base-pair and stacking restraints. Iterations of manual refinement (using Coot) and automated, energy-based refinement (using Rosetta/Phenix) resulted in the final atomic model.

$TnsB^{CTD}$-TnsC sample preparation and imaging for cryo-EM

TnsC was purified following previously described protocols (2, 3). A truncated TnsB construct, consisting of the C-terminal 109 residues from wild-type TnsB (termed hereafter as $TnsB^{CTD}$), was cloned from pXT129_TwinStrep-SUMO-ShTnsB vector (Addgene #135525) using Q5 site-

directed mutagenesis kit (NEB) and two primers: TnsB_Cter_R and TnsB_Cter_F (Table S2). $TnsB^{CTD}$ clones were transformed into E.Coli BL21-RIPL competent cells (Agilent) and purified using previously described protocols (2, 3).

To prepare samples for cryo-EM imaging, TnsC filaments were reconstituted by mixing purified TnsC with a 1/10 molar ratio of 22bp dsDNA (annealed using 22bp_top and 22bp_bottom, Table S2) in the following buffer: 25 mM HEPES pH 7.5, 2% glycerol, 1 mM DTT, 200 mM NaCl, 2 mM $MgCl_2$, and 2 mM AMP-PNP. TnsC filaments were incubated on ice for 5 minutes followed by adding a 2-fold molar excess of purified $TnsB^{CTD}$. The mixture was subsequently incubated for 30 minutes on ice prior to vitrification. The final concentration of TnsC, $TnsB^{CTD}$, and DNA in the final sample corresponded to 30 μM, 60 μM, and 3 μM respectively. Vitrification using the Mark IV Vitrobot (ThermoFisher) was carried out using the following chamber settings: 4 °C and 100% humidity. 4 μL of the reconstituted complex was loaded on a freshly glow discharged R1.2/1.3 grid (UltraAuFoil, Quantifoil) using an easiGlow unit (PELCO) with the following settings: 30 mA current for 30 seconds. Each grid was blotted for 7 seconds with blot force of 5 and immediately plunged into a slurry of ethane cooled with liquid nitrogen for vitrification. Grids were imaged using Talos Arctica (ThermoFisher) operated at 200 keV, which is equipped with a BioQuantum energy filter (Gatan) and K3 direct electron detector (Gatan). The microscope was aligned as described earlier. A total of 2,795 movies were collected using SerialEM (9), with 3 by 3 image shift at the nominal defocus from -1.0 μm to -2.5 μm, and 63,000X magnification (1.33 Å per pixel). Movies were fractionated into 50 frames, corresponding to a total dose of 50 electrons / $Å^2$ spread over 3.1 seconds.

$TnsB^{CTD}$-TnsC cryo-EM image processing

As described above, Warp (10) was used for beam-induced motion correction, CTF estimation, and initial particle picking for 2,795 collected movies. Warp particle stacks were exported to cryoSPARC (12) for 2D classification; curation of 2D class averages resulted in 699,656 particles. Applying a CTF-resolution cut-off of 4 Å resolution at this stage resulted in 356,489 particles, which were used for downstream refinement in cryoSPARC. A 20 Å low-pass filtered map of the ATPγS-bound TnsC filament (EMD-23720)(2) was used as an initial reference for helical refinement. Helical symmetry was locally optimized in cryoSPARC with a twist range: 53° – 63° and rise range: 6.147 Å – 7.513 Å. Refined helical parameters converged to a helical twist of 59.3° and a helical rise of 7 Å. Cryosparc particle stacks were exported to RELION(13, 22) for subsequent refinement. 3D classification without alignment resulted in one class containing high-resolution structural features, corresponding to 80% of the total particle number (286,988). The second round of helical refinement in RELION, followed by CTF refinement (14), resulted in the final cryo-EM map, estimated to be 3.5 Å resolution.

TnsB^{Hook}-TnsC sample preparation and imaging for cryo-EM

The TnsB^{Hook} peptide (residue 570-584) was chemically synthesized and HPLC-purified (AlanScientific). Lyophilized TnsB^{Hook} peptide was resuspended in 25 mM HEPES, 200 mM NaCl, 2% glycerol, and 1 mM DTT. TnsC filaments were polymerized by mixing purified TnsC with 1/25 molar ratio of 60bp dsDNA (annealed using 60bp_top and 60bp_bottom, Table S2), in the following buffer: 25 mM HEPES pH 7.5, 2% glycerol, 1 mM DTT, 200 mM NaCl, 2 mM $MgCl_2$, and 2 mM AMP-PNP. TnsC filaments were incubated on ice for 5 minutes followed by the addition of 4-fold molar excess of the TnsB^{Hook} peptide. The concentration of TnsC, TnsB^{Hook}, and DNA in the final sample (used for preparing cryo-EM grids) corresponds to: 26.5 μM, 106 μM, and 1 μM respectively. The sample was incubated for 10 minutes at 37°C before vitrification. Sample

vitrification and cryo-EM imaging were carried out as with the TnsB^{CTD}-TnsC sample (described above).

TnsB^{Hook}-TnsC cryo-EM image processing

2,056 collected movies were pre-processed using Warp (10) for beam-induced motion correction, CTF estimation, and particle picking. The Warp generated particle stack (873,674 particles) was imported to cryoSPARC (12) for downstream image processing. Selection based on 2D class averages resulted in 562,516 particles. Helical refinement was performed as previously described (see above, TnsB^{CTD}-TnsC reconstruction), which resulted in a cryo-EM reconstruction with estimated 3.77 Å resolution. Final helical parameters corresponded to 59.3° helical twist, and 7 Å helical rise, identical to the previously determined TnsB^{CTD}-TnsC helical reconstruction.

Image-based TnsC Filament Disassembly Assay

All the TnsB truncation constructs were cloned using pXT129_TwinStrep-SUMO-ShTnsB vector (Addgene #135525) and Q5 site-directed mutagenesis kit (NEB). C-terminal truncation constructs (TnsB$^{\Delta Hook}$, TnsB$^{\Delta CTD}$) were purified using previously described protocols(2, 3). The TnsB^{Hook} peptide was prepared using chemical synthesis (AlanScientific, see above). Both TnsC and 60 bp dsDNA (Table S2) (25:1 molar ratio) were diluted into the following reconstitution buffer: 2 mM nucleotide (ATP or AMP-PNP, Sigma-Aldrich), 25 mM HEPES, 200 mM NaCl, 2% glycerol, 1 mM DTT, and 2mM $MgCl_2$. Filaments were then either incubated with a 1:1 molar ratio of TnsB constructs or an equivalent volume of reconstitution buffer (negative control). Final concentrations of TnsB, TnsC, and DNA are 8 μM, 8 μM, and 320 nM, respectively. Reaction mixtures were incubated at 30°C for one hour, followed by negative stain electron microscopy imaging (EM). 4 μL of 3-fold diluted sample was incubated on a continuous carbon grid (Electron Microscopy Sciences) before washing 3 times in 3 x 50 μL droplets of Milli-Q water. Grids were then washed

3 times in 3 x 50 µL droplets of freshly filtered (0.22 µm syringe filter) 2% Uranyl Acetate stain. Images were collected on a 120 keV BioTwin (FEI) at a magnification of 135,000x, corresponding to a scaling of 2.40 Å per pixel.

TnsC Filament Disassembly Pull Down Assay

Pull down assays were performed using Streptavidin Mag Sepharose magnetic beads (Cytiva). The beads were first washed with reconstitution buffer: 2 mM ATP, 25 mM HEPES, 200 mM NaCl, 2% glycerol, 1 mM DTT, and 2mM $MgCl_2$, followed by 0.1 mg/mL BSA washes to prevent non-specific binding. A 74 bp desthiobiotinylated dsDNA substrate was made by annealing a 14 bp desthiobiotinylated oligonucleotide (LUEGO (23), Table S2) with two DNA oligos (60bp_bottom and 74bp_top, Table S2). 20 µL of the 74 bp dsDNA mixture (corresponding to a concentration of 30 µM) was incubated with 20 µL of magnetic beads followed by multiple washes to remove excess DNA substrate. 50 µL of 32 µM TnsC was added to the DNA-bound beads along with 1 mM ATP or AMP-PNP to initiate filament formation. Multiple washes with reconstitution buffer were used to remove excess TnsC before adding TnsB. Next, 20 µL of 80 µM full-length TnsB or TnsB truncation constructs: $TnsB^{\Delta Hook}$, $TnsB^{\Delta CTD}$, $TnsB^{Hook}$, or $TnsB^{CTD}$ were incubated with the magnetic beads at 30 °C for one hour. Extensive washes were used to remove excess TnsB and disassembled TnsC prior to elution. DNA was incubated with 20 µL of reconstitution buffer supplemented with 4 mM Biotin for 10 minutes at 37 °C prior to collecting the eluate for analysis.

V-K CAST family multiple sequence alignment

167 V-K CAST elements containing both the TnsB+TnsC+TniQ operon and Cas12k genes were identified from all the annotated NCBI cyanobacterial genomes. TnsB protein sequences from the identified elements were made non-redundant at a 90% sequence identity cutoff. Representative TnsB sequences were chosen based on the following criteria: well-defined transposon ends,

fewer pseudogenes on core transposon genes, and intact target-site duplication. The resulting pool of TnsB representative sequences, including the ShTnsB sequence were subjected to MUSCLE sequence alignment (24) through the EMBL-EBI server (25).

Model validation

Model-map Fourier Shell Correlation (FSC) of the refined atomic models were calculated using the comprehensive validation tool in Phenix graphical user interface (20). Model-map FSC resolution was estimated from the model-map FSC curve using a 0.5 cutoff. Validation statistics, including: MolProbity score (26), clashes between atoms, Ramachandran plot, bond-length/angle, and side-chain rotamers were obtained using the comprehensive validation tool in Phenix(27). EMRinger (28) was run using the Phenix GUI (20) for all the deposited models. To validate anisotropy of the reconstruction, 3DFSC was run using the 3DFSC server (29). All validation statistics are summarized in Table S1.

Movie S1. Architecture of TnsB Strand-transfer complex explained.

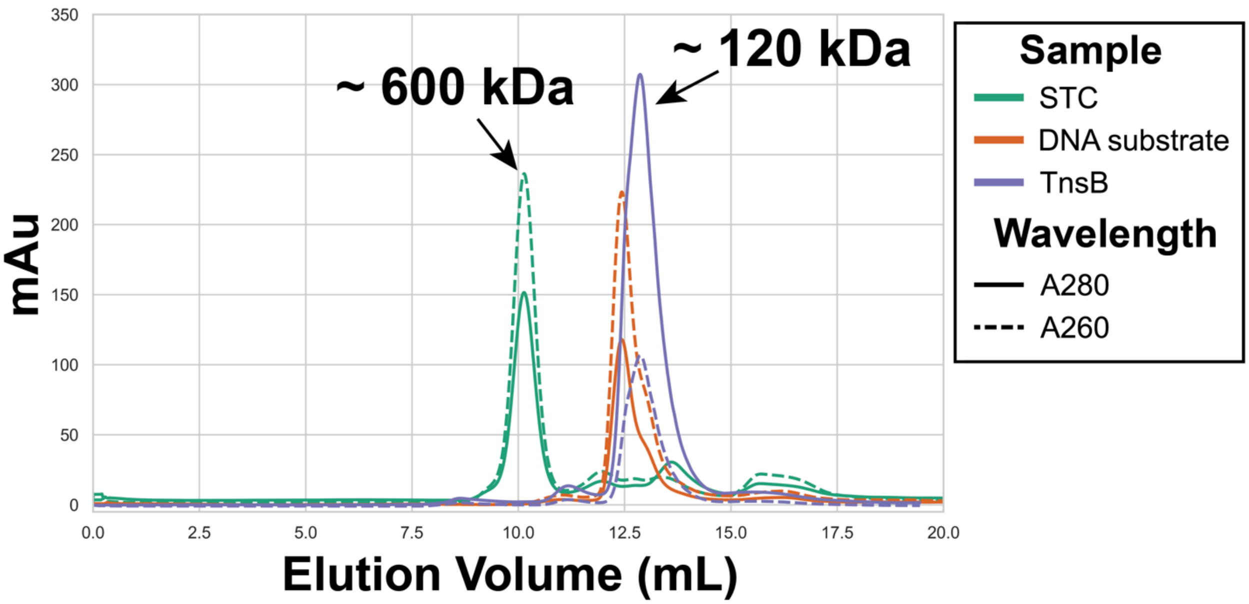

Figure S1. Size-exclusion chromatography identifies a large protein-DNA complex when TnsB is incubated with the designed strand-transfer DNA substrate. The reconstituted TnsB strand-transfer complex (STC, green), DNA substrate (orange), and TnsB (purple) were subjected to size exclusion chromatography. Three separate runs are overlaid for comparison. Absorbance at 280 nm and 260 nm are represented using solid and dashed lines, respectively. Elution volumes (mL) are shown for a Superdex S200 increase 10/300 GL. Molecular weights corresponding to the elution volumes of STC or TnsB monomers are reported for each peak.

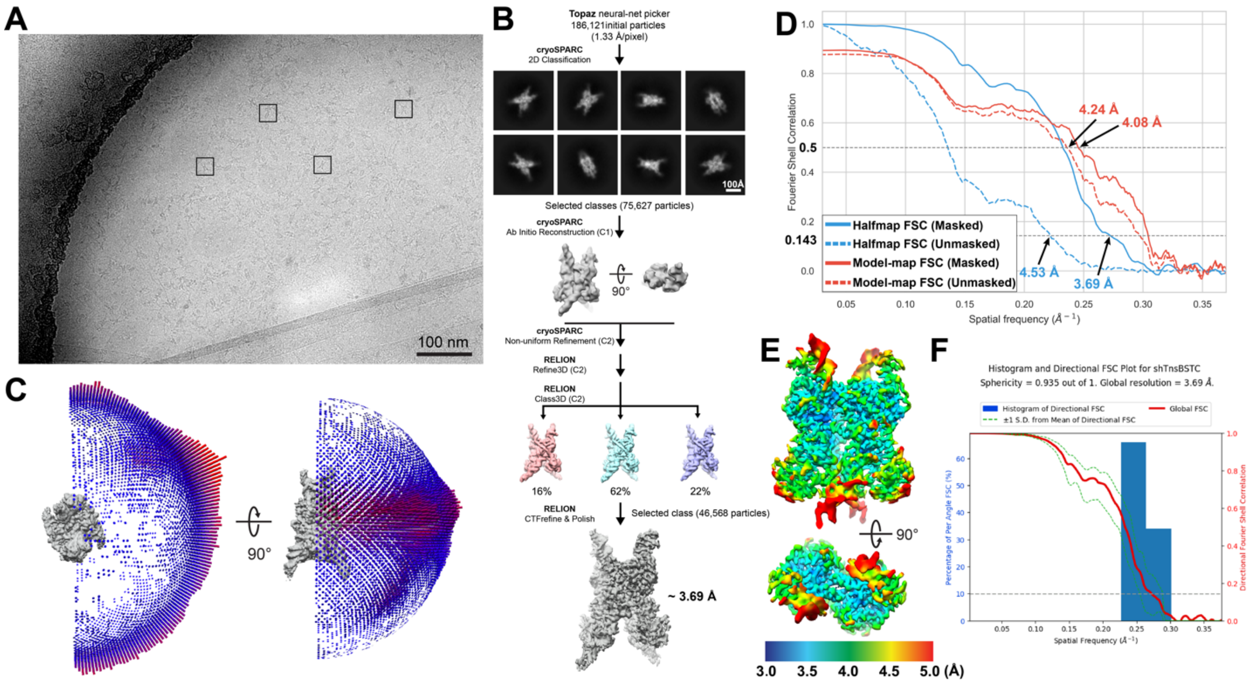

Figure S2. Cryo-EM imaging and image processing pipeline for the TnsB strand-transfer complex (STC). A. Representative micrograph from the TnsB STC dataset. Multiple layers of GO can be seen in the bottom right-hand corner of the image. Selected particles are indicated with a black box. Scale bar (black, bottom right) represents 100 nm. **B.** Image processing workflow used to analyze the TnsB STC dataset. Unbiased 2D classifications and *ab initio* reconstruction resulted in a C2 symmetric assembly. All subsequent processing steps enforced C2 symmetry. 3D classification resulted in the colored volumes shown (pink, blue, and purple). The most populated class (62% of particles) was refined to produce the final 3D reconstruction shown at the bottom. **C.** Euler angle histogram of the final C2 symmetric reconstruction. Colors indicate counts; red corresponds to high particle counts for that particular viewing angle, blue to low particle counts. **D.** Fourier shell correlation (FSC) curve of TnsB STC complex. Masked (solid) or unmasked (dashed) gold standard half-map FSC (blue) and model-map FSC (red) curves are shown for the TnsB STC refined reconstruction and atomic model. Appropriate cut-offs (0.5 for model-map and 0.143 for half-maps) are indicated with dashed lines and estimated resolution is indicated accordingly. **E.** Local resolution filtered reconstruction is shown with estimated local resolution values (colors). Legend at the bottom indicates local resolution range and values in Angstrom. **F.** Histogram of directional FSC. Directional FSC is estimated using 3DFSC server (29), and ranges from 3.4 Å to 4.3 Å. The blue bar graph shows a histogram of the estimated resolution range for orthogonal directions. The red curve represents global (i.e. directionless) FSC. The green curve indicates +/- 1 standard deviation (S.D.).

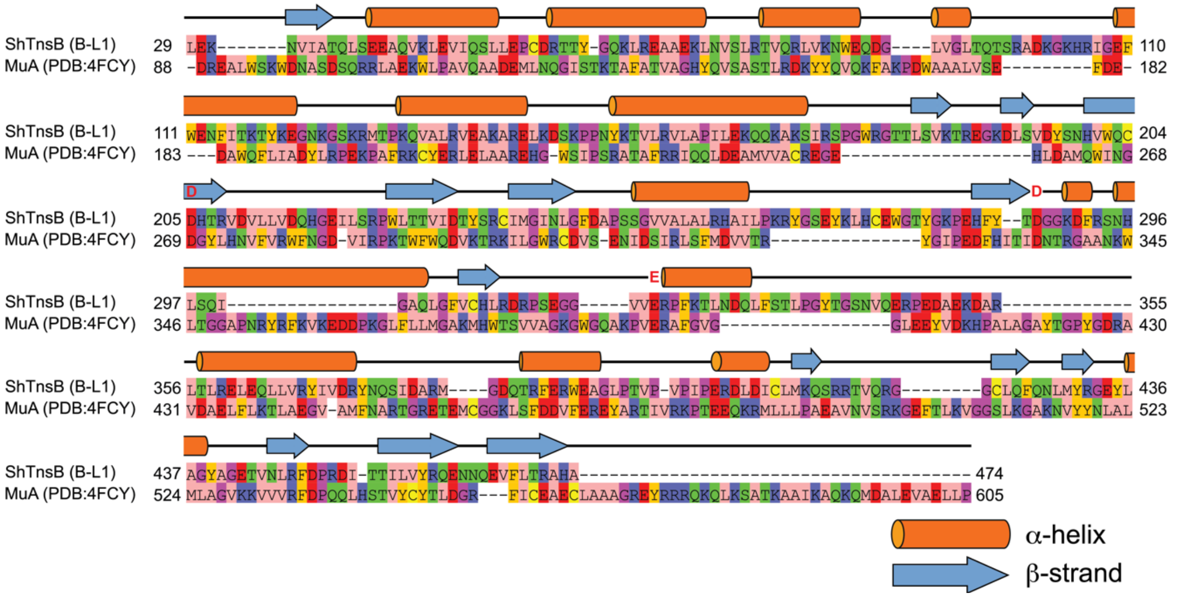

Figure S3. Structure-based sequence alignment of ShCAST TnsB and MuA, shown with TnsB secondary structure assignments. Structure-based sequence alignment of ShCAST TnsB (ShTnsB) B-L1 subunit and R2-bound MuA subunit (PDB: 4FCY, chain B) was generated using PROMALS3D (30). Alpha-helices are displayed as orange cylinders, beta-strands are displayed as blue arrows. The conserved DDE catalytic residues are indicated with red characters on the secondary structure diagram, positioned just above the sequence alignment.

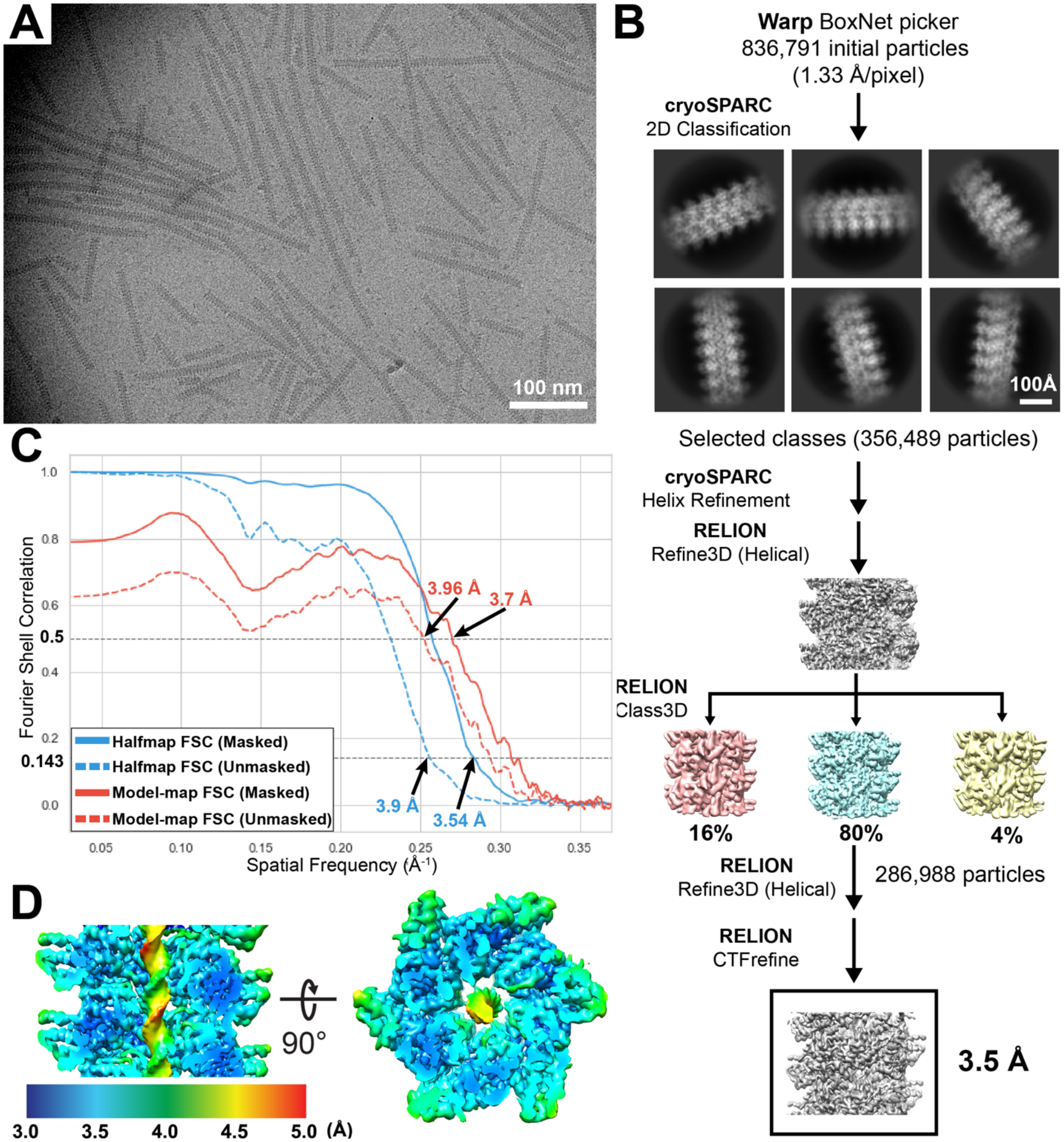

Figure S4. Image processing pipeline for TnsB^{CTD}-TnsC filament cryo-EM reconstruction. **A.** Representative micrograph from the TnsB^{CTD}-TnsC dataset. White scale bar represents 100 nm. **B.** Image processing workflow from pre-processing (top) to final TnsB^{CTD}-TnsC filament reconstruction (boxed at the bottom). 2D classes are shown with 100 Å scalebar. The output of 3D classification is shown below in different colors (pink, blue, and yellow). **C.** Fourier-shell correlation (FSC) curve for masked (solid) and unmasked (dashed) refined reconstruction (blue is gold-standard FSC) and model-map FSC (red). 0.5 cutoff (dashed lines) indicates the standard cutoff for estimating model-map resolution, whereas 0.143 cutoff indicates the standard cutoff for estimating the resolution of the cryo-EM reconstruction. **D.** Local resolution (colors) is

shown mapped onto the cryo-EM map, ranging from 3 - 5 Å local resolution. Legend at the bottom indicates local resolution values in Angstrom.

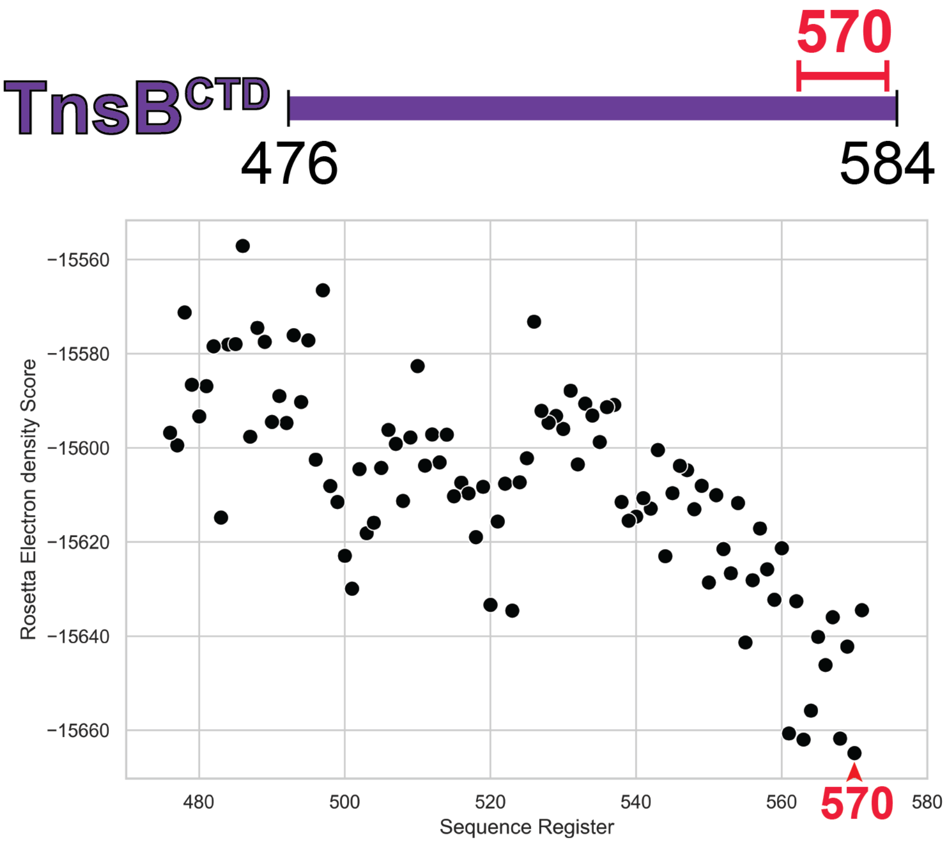

Figure S5. Rosetta modeling identifies an optimal sequence register for the observed TnsB^{Hook} cryo-EM density. Rosetta modeling (see Materials and Methods for details) was used to identify the sequence register that best describes the observed density. Each possible sequence register is represented by a black data-point, plotted according to the starting position on the x-axis. The Rosetta energy of the modeled fragment is plotted on the y-axis. The optimal sequence register is identified by the lowest-scoring datapoint, corresponding to the fragment beginning at position 570. The domain diagram of the C-terminal construct imaged (purple line) spans residues 476-584. The optimal sequence register of the observed fragment corresponds to residues 570-583. Full-length TnsB has 584 residues.

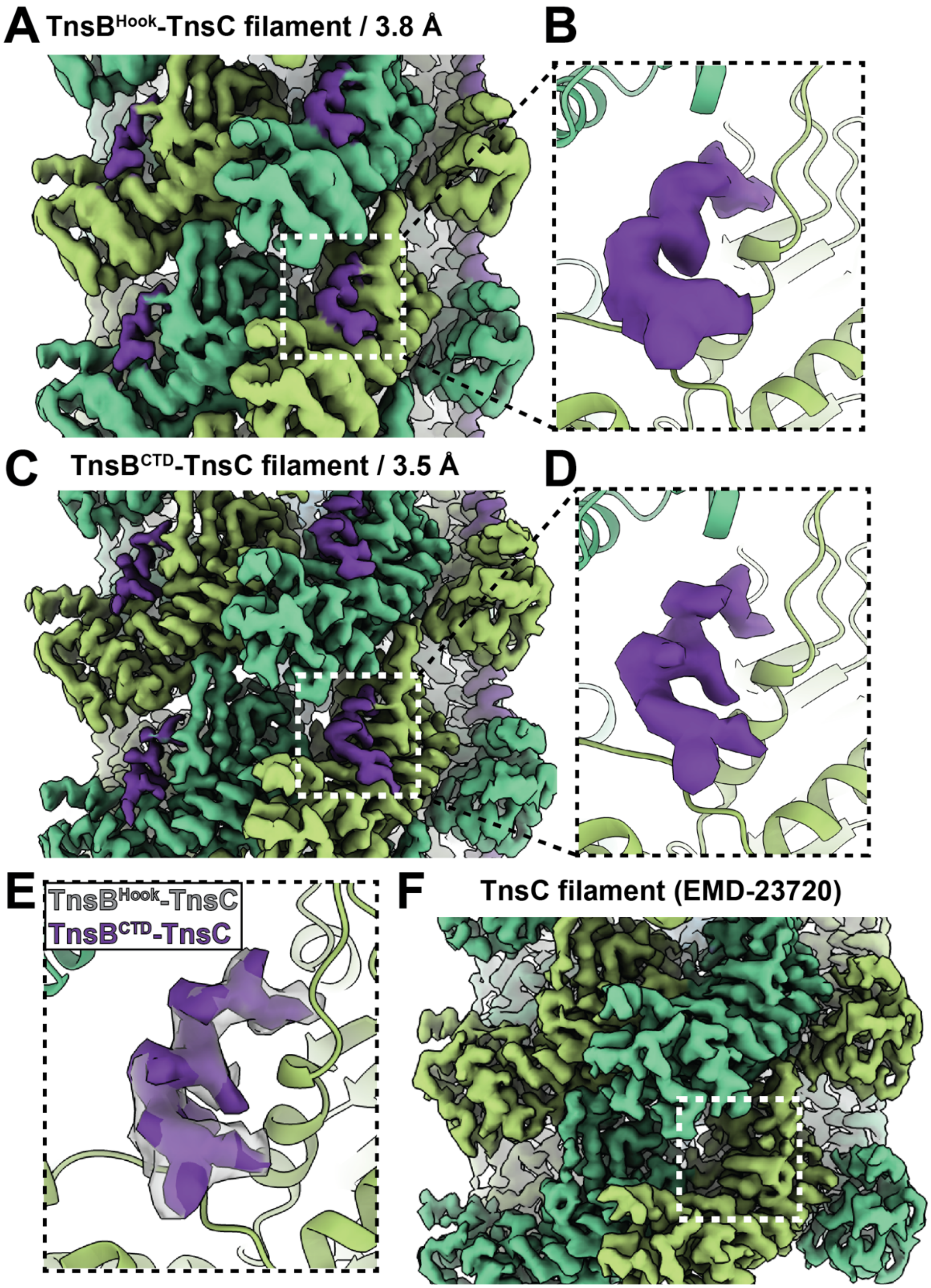

Figure S6. Cryo-EM reconstruction of TnsB^{Hook} is indistinguishable from the cryo-EM reconstruction of TnsB^{CTD} A. 3.8 Å resolution cryo-EM reconstruction of TnsB^{Hook} (purple) bound to TnsC filament (green). White dashed box indicates region visualized in panel B**.** Close-up view of the segmented cryo-EM density of TnsB^{Hook} from TnsB^{Hook}-TnsC reconstruction. **C.** Cryo-EM reconstruction of TnsB^{CTD} bound TnsC (3.5 Å average resolution). White dashed box indicates the region for close-up view in panel D. **D.** Zoomed-in view of TnsB^{Hook} reconstruction segmented from the TnsB^{CTD}-bound TnsC filament. **E.** Overlaid cryo-EM densities of the

segmented densities from the TnsB^{Hook}-TnsC reconstruction (semi-transparent grey) and TnsB^{CTD}-TnsC reconstruction (purple). **F.** Cryo-EM reconstruction of TnsC filament (EMD-23720) for comparison purposes. White dashed box indicates TnsB^{Hook} binding site.

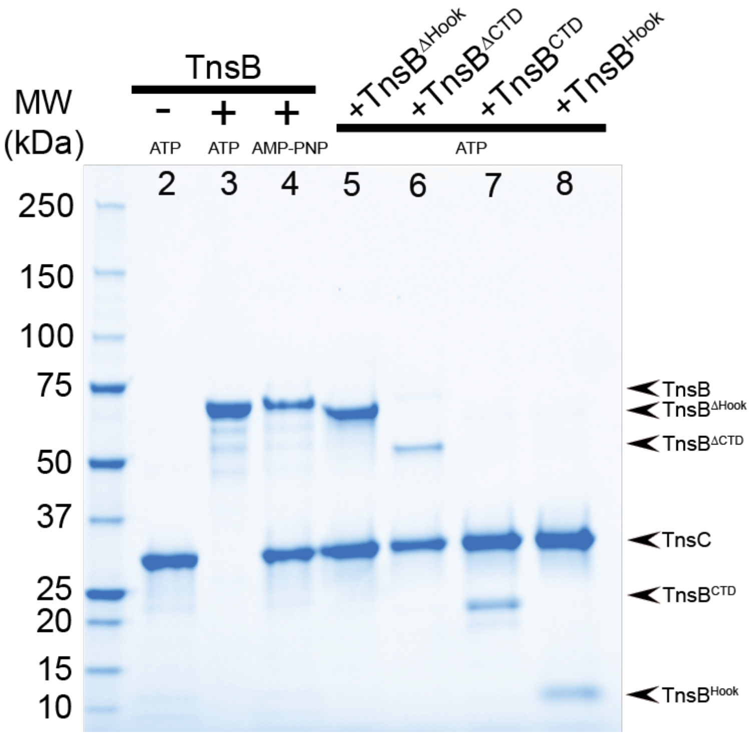

Figure S7. Biochemical pull-downs demonstrate that none of the TnsB truncation constructs disassemble TnsC filaments. We used DNA-based pulldowns to assess whether TnsC filaments can form on double-stranded DNA. TnsC filaments were first reconstituted on the streptavidin bead-bound DNA in the presence of ATP or AMP-PNP. Either full-length TnsB (lanes 2-4, + and – symbol indicates whether TnsB was added or not) or TnsB truncations (lanes 5-8, naming follows conventions established in Figure 2A) were assessed for their ability to stimulate TnsC filament disassembly at equivalent molar concentration (see materials and methods for details). The expected position of each protein is indicated on the right side of the gel. Wild-type TnsB disassembles ATP-bound TnsC filaments (lane 3), but doesn't disassemble AMP-PNP bound TnsC filaments (lane 4). None of the truncation constructs were able to disassemble ATP-bound TnsC filaments.

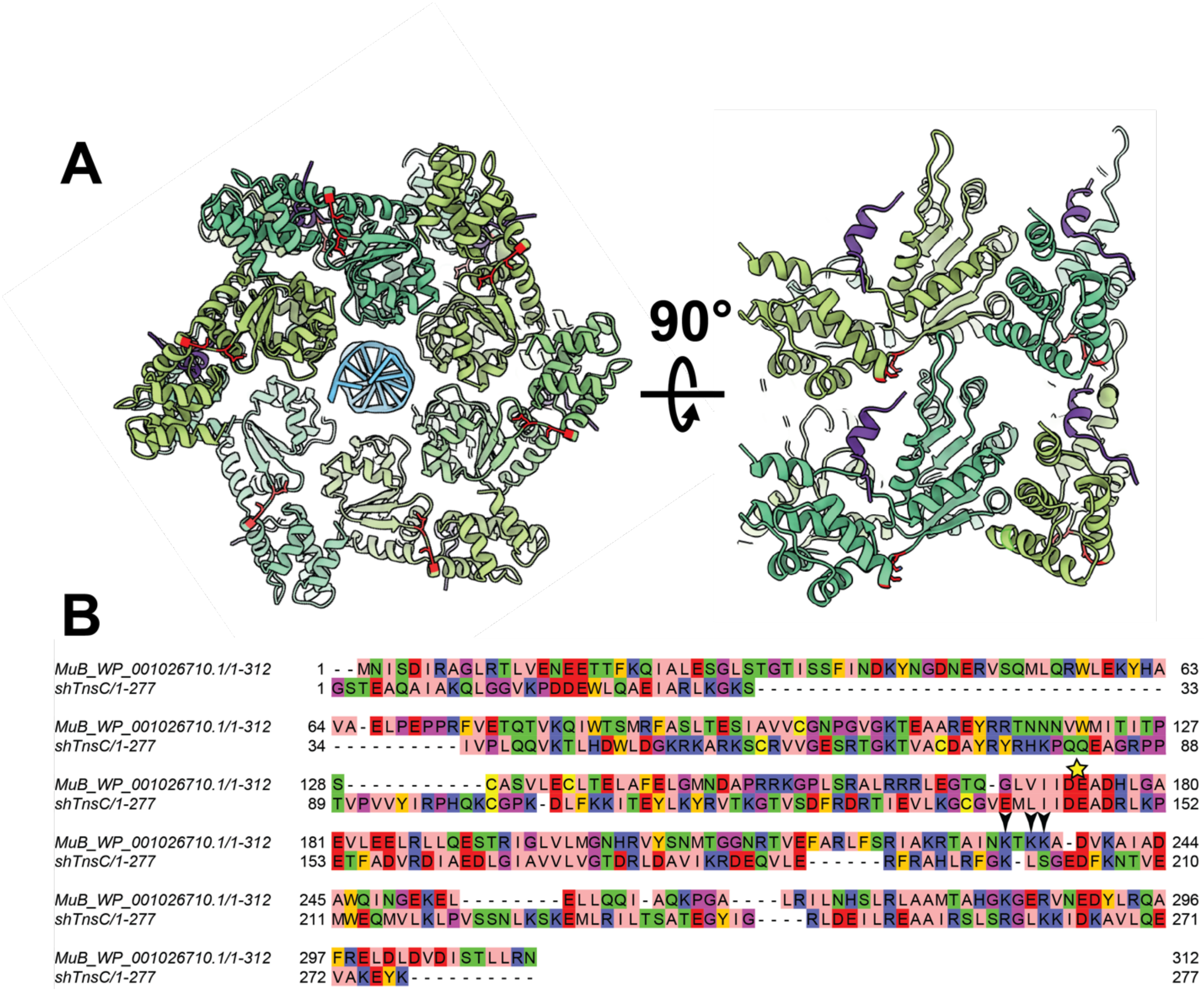

Figure S8. The equivalent positions on TnsC corresponding to the MuA-MuB interaction interface localize close to the TnsB^{Hook} binding site. A. The equivalent positions previously identified as the site of interaction between MuA and MuB (K233, K235, and K236 in MuB) are colored red on the ShCAST TnsC filament (TnsC is green, DNA is blue). The location of the TnsB^{Hook} fragment is shown in purple on the TnsC filament side view (right). **B.** Sequence alignment of MuB and TnsC. Black arrows indicate the equivalent position where the lysine residues in MuB interact with MuA (31), and the yellow star indicates the conserved Walker B motif.

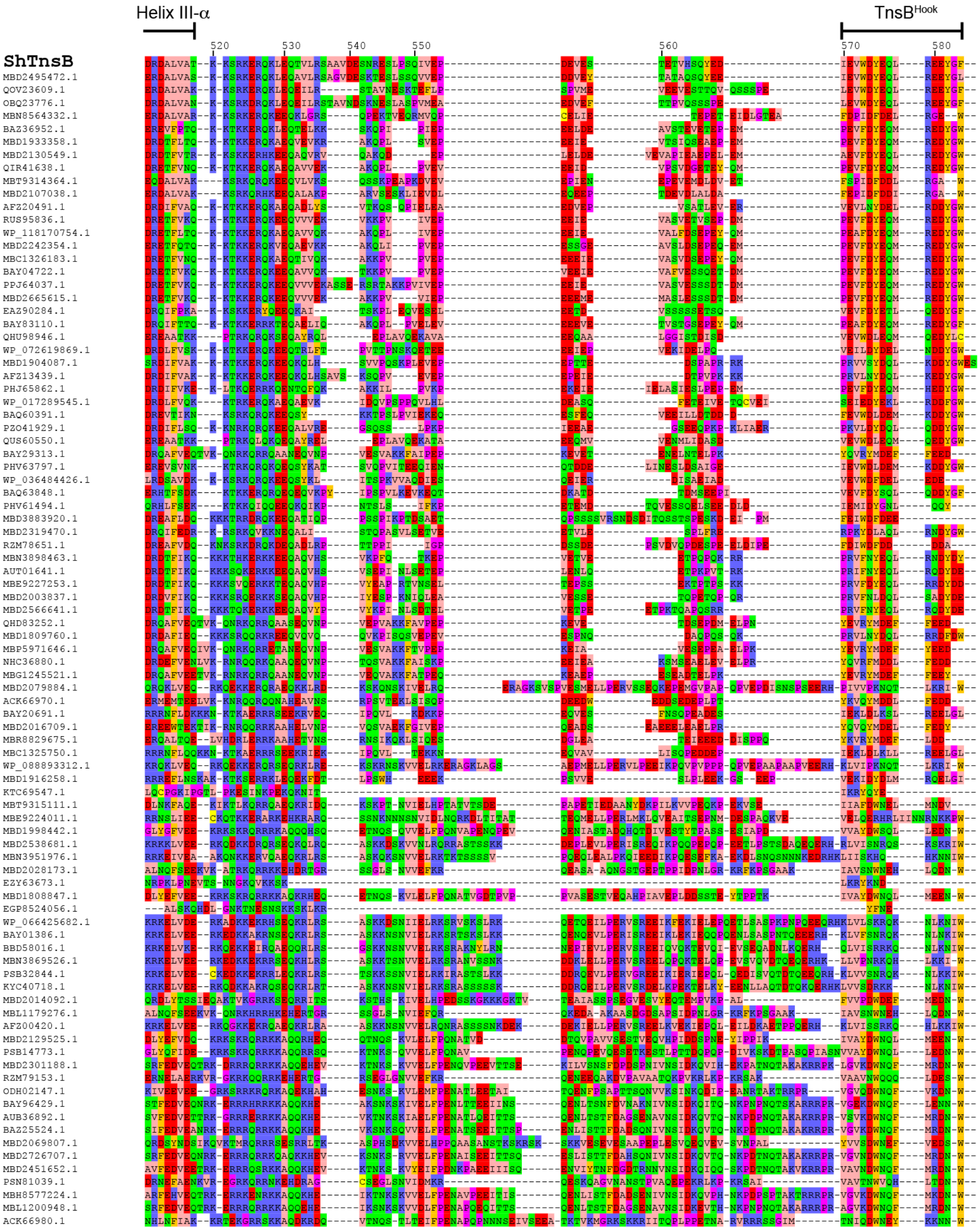

Figure S9. Sequence alignment of the TnsB homologs from the V-K CAST subfamily, focusing on the variability within the C-terminus. ShCAST TnsB is the first sequence in a group of homologous TnsB proteins from the V-K CAST family, shown are positions 512 - 584.

Domains are indicated at the top. Residues are colored according to biochemical property. Homologs are labeled by Genbank accession id.

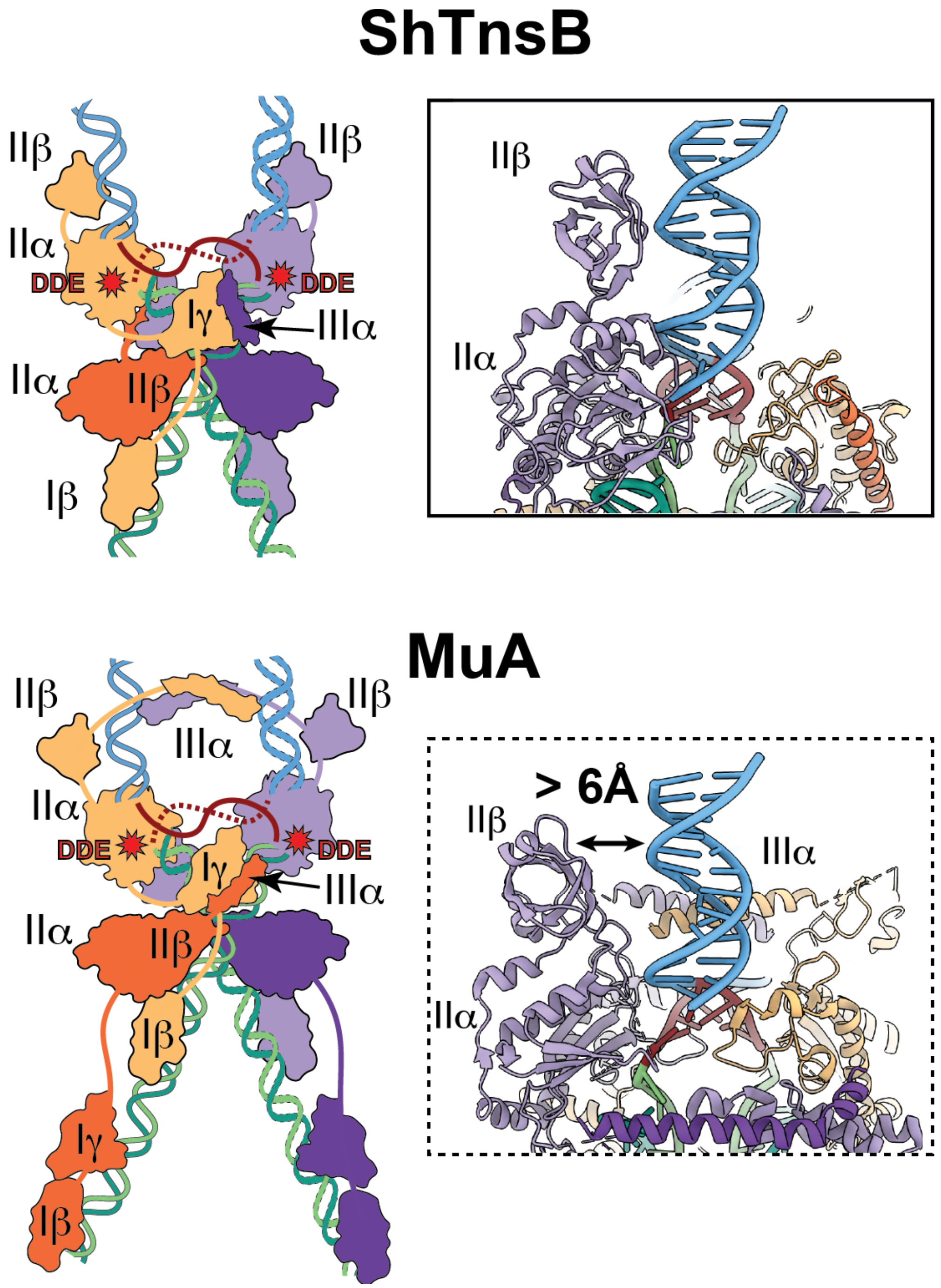

Figure S10. TnsB domain IIβ is positioned to stabilize target-DNA. Architectural diagrams for ShCAST TnsB (ShTnsB) and MuA (left) are included as a reference, copied from Figure 1F & 1G. Domain IIβ in both MuA and ShTnsB are positioned close to target-DNA (blue). In the case of ShTnsB, IIβ(light purple, top-right panel) is close enough to interact with the target DNA. In MuA (bottom right panel corresponds to PDB 4FCY), domain IIβ is more than 6 Å away from target-DNA (indicated with a black double arrow).

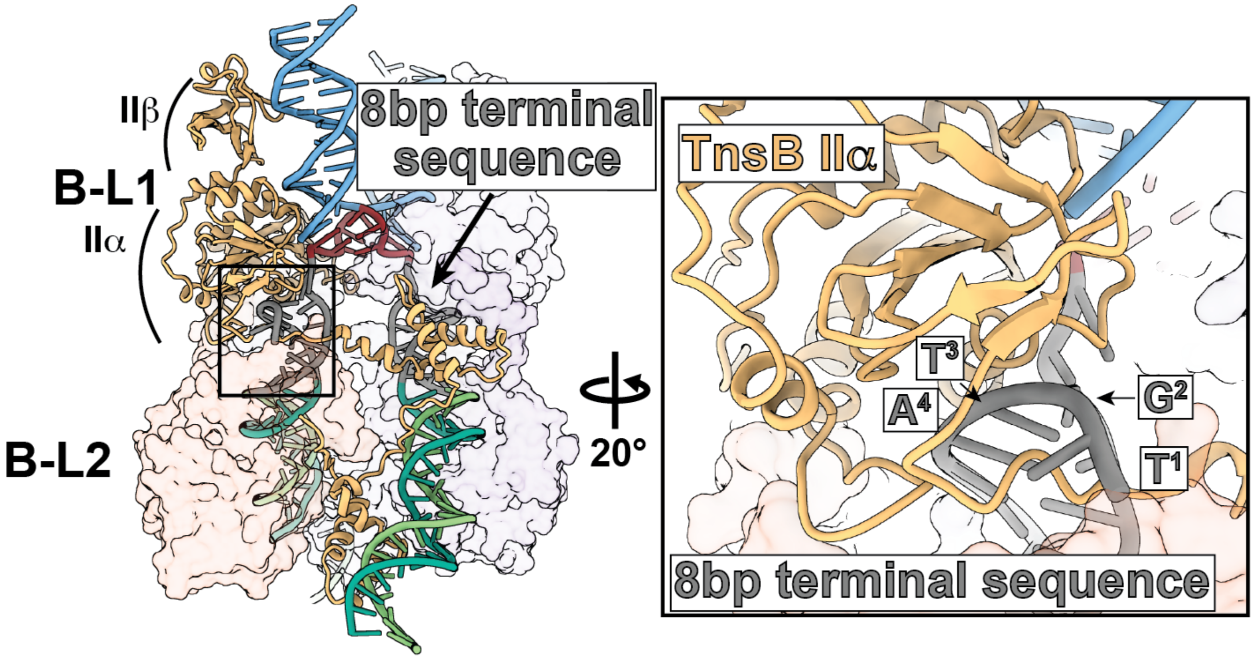

Figure S11. The TnsB catalytic domain (IIα) is interacts with the 8 base pair terminal sequence. An overview of the TnsB STC structure (left) is shown, with transparent surface for 3 of the 4 TnsB subunits. The B-L1 TnsB monomer (tan) is shown in ribbon to highlight its interactions with the 8 base-pair terminal sequence (gray). target-DNA is colored blue, and the TnsB binding site is colored green. The inset on the left corresponds to a zoomed in version of a slightly rotated view (right). Nucleotide positions indicate positions on the non-transferred strand visualized in the inset.

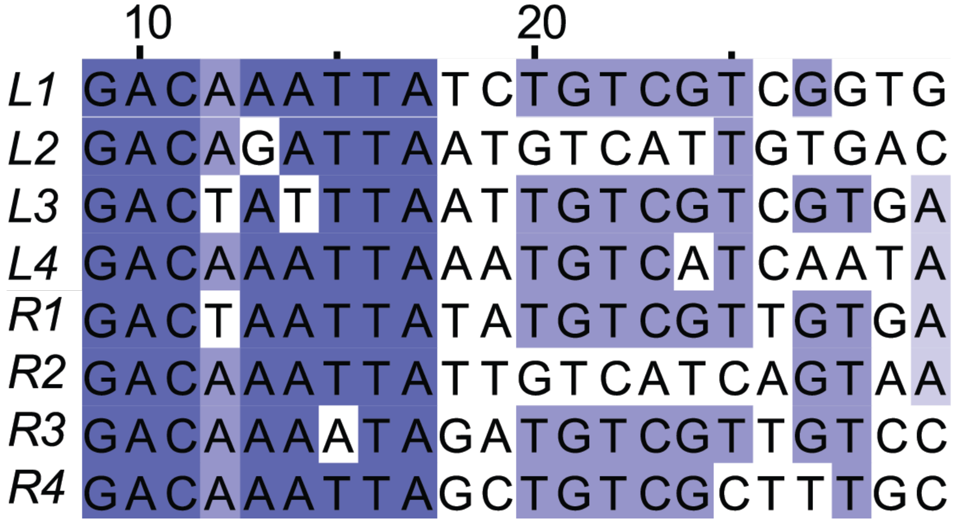

Figure S12. Multiple sequence alignment of TnsB binding sites from the ShCAST transposon ends. Each of the four TnsB binding site from the left (L1 - L4) and right (R1 - R4) ends of the non-transferred strand are shown aligned in the 5' to 3' direction. Nucleotides are colored according to sequence conservation; darker colors represent higher sequence conservation whereas lighter colors represent low sequence conservation.

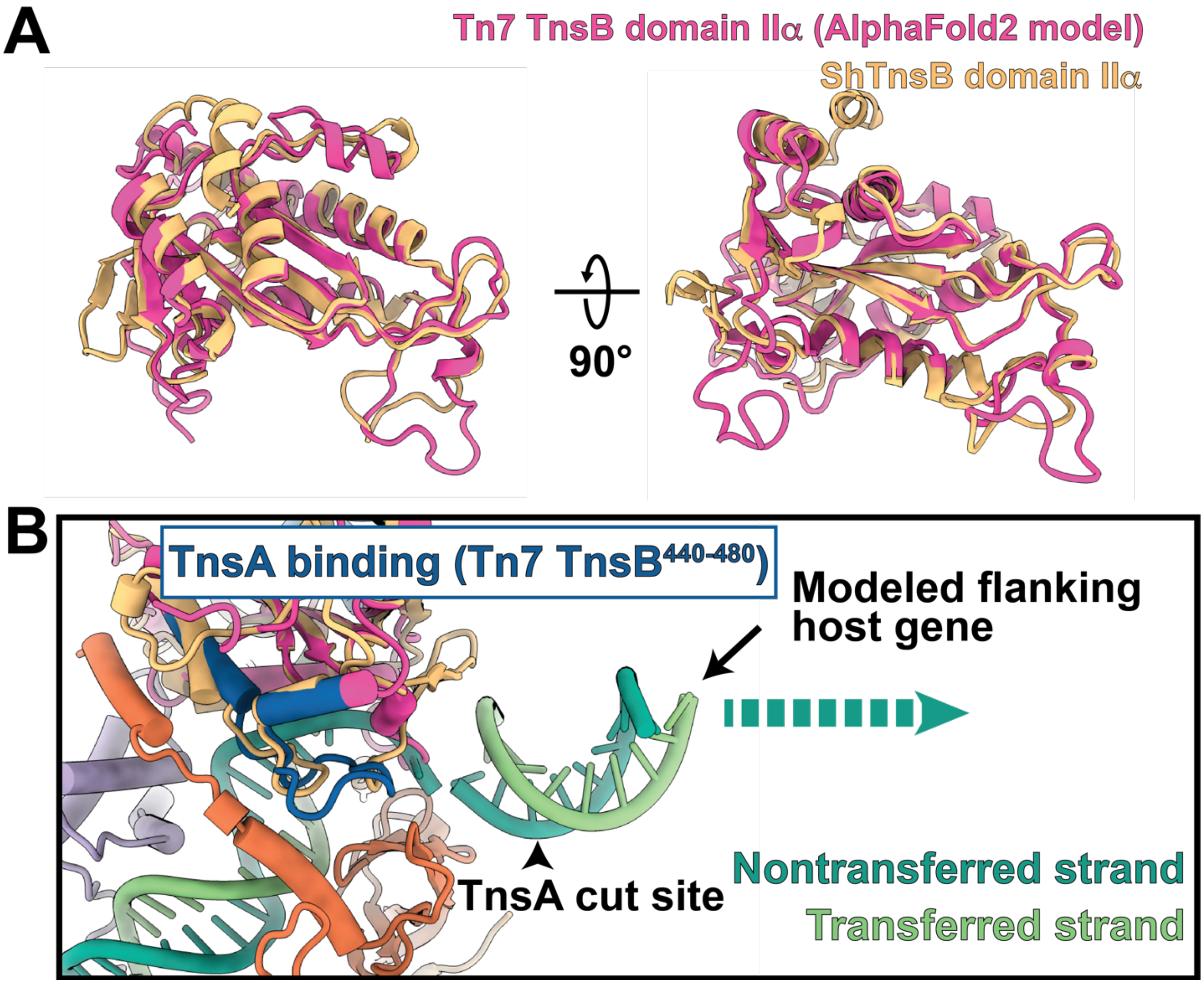

Figure S13. The predicted location of TnsA-TnsB interactions for prototypic Tn7, based on the ShCAST TnsB STC structure, is close to the expected location of TnsA cleavage on the non-transferred strand. A. Using AlphaFold2, we predicted the structure of prototypic Tn7 TnsB (pink), which closely matches the experimentally determined structure of ShCAST TnsB domain IIα (tan). The measured global RMSD is 2.4 Å. **B.** The AlphaFold2 predicted structure (pink) is shown overlaid onto domain IIα of TnsB (B-L1) in the context of the STC atomic model (tan, orange, purple). The location of flanking DNA is shown adjacent to the non-transferred strand and its direction is indicated with dashed arrows. The experimentally mapped TnsA binding site on prototypic Tn7 TnsB (residues 440-480) is colored dark blue. This localizes very close to where the predicted TnsA cut site would be (indicated with a black arrow).

Name	TnsB^{CTD}-TnsC filament	TnsB strand-transfer complex	TnsB^{Hook}-TnsC filament
PDB ID	7SVV	7SVW	-
EMDB ID	EMD-25454	EMD-25455	EMD-27140
**Data collection and Processing**			
Microscope	Talos-Arctica	Talos-Arctica	Talos-Arctica
Voltage (keV)	200	200	200
Camera	K3	K3	K3
Magnification	63,000	63,000	63,000
Pixel size at detector (Å/pixel)	1.33	1.33	1.33
Total electron exposure (e^-/Å^2)	50	49	50
Exposure rate (e-/pixel/sec)	28.3	28.9	26.8
Number of frames	50	49	50
Defocus range (µm)	-1 – -2.5	-1 – -2.5	-1 – -2.5
Automation software	SerialEM	SerialEM	SerialEM
Energy filter slit width	20 keV	20 keV	20 keV
Micrographs collected (no.)	2,795	1,925	2,056
Micrographs used (no.)	1,392	1,874	2,029
Total extracted particles (no.)	836,791	186,121	873,674
**For each reconstruction:**			
Refined particles (no.)	356,489	75,627	562,516
Final particles (no.)	286,988	46,568	562,516
Symmetry	Helical	C2	Helical
Resolution (global, Å)			
FSC 0.5 (unmasked/masked)	4.37/3.91	7.39/4.34	4.56/4.04
FSC 0.143 (unmasked/masked)	3.91/3.54	4.53/3.69	4.16/3.77
Resolution range (local, Å)	3.5 – 5	3.5 – 5	3.5 – 5
Resolution range due to anisotropy (Å)	-	3.4 – 4.2	-
Map sharpening *B* factor (Å^2)	-131.2	-28.7	-149.1
Map sharpening methods	RELION	RELION	cryoSPARC
**Model composition**			
Protein residues	2,710	1,484	-
Ligands	20	2	-
RNA/DNA	38	190	-
**Model Refinement**			
Refinement package	Coot/Rosetta/Phenix	Coot/Rosetta/Phenix	-
- real or reciprocal space	Real space	Real space	-
- resolution cutoff (Å)	3.5	3.7	-
Model-Map scores			
- CC	0.77	0.72	-
- Average FSC (0.5 cutoff, Å)	3.70	4.07	-
R.m.s deviations from ideal values			
Bond length (Å)	1.836	1.426	-
Bond angles (°)	0.019	0.011	-
**Validation**			
MolProbity score	1.22	2.29	-
CaBLAM outliers (%)	0.76	2.22	-
Clashscore	4.47	14.27	-
Poor rotamers (%)	0.00	2.78	-
C-beta outliers (%)	0.39	0.65	-

EMRinger score	1.85	1.47	-
Ramachandran plot			
Favored (%)	98.58	95.77	-
Outliers (%)	0.00	0.27	-

Table S1. Cryo-EM data collection, refinement, and validation statistics.

Oligonucleotides

Name	Description	Sequence	Used Figure(s)
LE_STC_For	Reconstitute strand transfer product, forward strand	ATGACATTAATCTGTCACCGACGACAGATAATTTGTCACTGTACAGGCCCTAGGTCTACGGTTAGAGGCT	1
LE_STC_Rev1	Reconstitute strand transfer product, reverse strand 1	TGTACAGTGACAAATTATCTGTCGTCGGTGACAGATTAATGTCAT	1
LE_STC_Rev2	Reconstitute strand transfer product, reverse strand 2	AGCCTCTAACCGTAGACCTA	1
TnsB_Cter_R	Cloning $TnsB^{CTD}$, reverse primer	GGATCCACCAATCTGTTCTCTGTG	2
TnsB_Cter_F	Cloning $TnsB^{CTD}$, forward primer	GGACTCGAAACAGAACAACTGGC	2
22bp_top	Substrate for reconstitution of $TnsB^{CTD}$-TnsC-TniQ	GCTTGAAGCGGCGCACGAAAAACGC	2C-D
22bp_bottom	Substrate for reconstitution of $TnsB^{CTD}$-TnsC-TniQ	AACGCTTTCGCGTTTTTCGTGCGCCGCTTCA	2C-D
60bp_top	Substrate for fast reconstitution of TnsC filaments	CGACAGCTCCTCCATGAAAGCAGTGGCCTTATTAAATGACTTCTCAACCAGTCAGCACGC	2F
60bp_bottom	Substrate for fast reconstitution of TnsC filaments	GCGTGCTGACTGGTTGAGAAGTCATTTAATAAGGCCACTGCTTTCATGGAGGAGCTGTCG	2F, S7
LUEGO	Substrate for DNA-based pull down assay	/5desBio/GTGCCCTGGTCTGG	S7
74bp_top	Substrate for DNA-based pull down assay	CGACAGCTCCTCCATGAAAGCAGTGGCCTTATTAAATGACTTCTCAACCAGTCAGCACGCCCAGACCAGGGCAC	S7

Table S2. Oligonucleotides used in this study

References

1. G. E. Ghanim, E. H. Kellogg, E. Nogales, D. C. Rio, Structure of a P element transposase-DNA complex reveals unusual DNA structures and GTP-DNA contacts. *Nat Struct Mol Biol* **26**, 1013-1022 (2019).
2. J. U. Park *et al.*, Structural basis for target site selection in RNA-guided DNA transposition systems. *Science* **373**, 768-774 (2021).
3. J. Strecker *et al.*, RNA-guided DNA insertion with CRISPR-associated transposases. *Science* **365**, 48-53 (2019).
4. D. C. Marcano *et al.*, Improved synthesis of graphene oxide. *ACS Nano* **4**, 4806-4814 (2010).
5. F. Wang *et al.*, General and robust covalently linked graphene oxide affinity grids for high-resolution cryo-EM. *Proc Natl Acad Sci U S A* **117**, 24269-24273 (2020).
6. D. T. Avinash Patel, Audrey Litvak, and Eva Nogales, Efficient graphene oxide coating improves cryo-EM sample preparation and data collection from tilted grids. *bioRxiv* https://doi.org/10.1101/2021.03.08.434344 (2021).
7. R. S. Pantelic, J. C. Meyer, U. Kaiser, W. Baumeister, J. M. Plitzko, Graphene oxide: a substrate for optimizing preparations of frozen-hydrated samples. *J Struct Biol* **170**, 152-156 (2010).
8. M. A. Herzik, Jr., Setting Up Parallel Illumination on the Talos Arctica for High-Resolution Data Collection. *Methods Mol. Biol.* **2215**, 125-144 (2021).
9. D. N. Mastronarde, Automated electron microscope tomography using robust prediction of specimen movements. *J Struct Biol* **152**, 36-51 (2005).
10. D. Tegunov, P. Cramer, Real-time cryo-electron microscopy data preprocessing with Warp. *Nat Methods* **16**, 1146-1152 (2019).
11. T. Bepler *et al.*, Positive-unlabeled convolutional neural networks for particle picking in cryo-electron micrographs. *Nat Methods* **16**, 1153-1160 (2019).
12. A. Punjani, J. L. Rubinstein, D. J. Fleet, M. A. Brubaker, cryoSPARC: algorithms for rapid unsupervised cryo-EM structure determination. *Nat Methods* **14**, 290-296 (2017).
13. S. H. Scheres, RELION: implementation of a Bayesian approach to cryo-EM structure determination. *J Struct Biol* **180**, 519-530 (2012).
14. J. Zivanov *et al.*, New tools for automated high-resolution cryo-EM structure determination in RELION-3. *Elife* **7** (2018).
15. J. Zivanov, T. Nakane, S. H. W. Scheres, A Bayesian approach to beam-induced motion correction in cryo-EM single-particle analysis. *IUCrJ* **6**, 5-17 (2019).
16. J. Jumper *et al.*, Highly accurate protein structure prediction with AlphaFold. *Nature* **596**, 583-589 (2021).
17. E. F. Pettersen *et al.*, UCSF Chimera--a visualization system for exploratory research and analysis. *J Comput Chem* **25**, 1605-1612 (2004).
18. P. Emsley, B. Lohkamp, W. G. Scott, K. Cowtan, Features and development of Coot. *Acta Crystallogr. D Biol. Crystallogr.* **66**, 486-501 (2010).
19. A. Leaver-Fay *et al.*, ROSETTA3: an object-oriented software suite for the simulation and design of macromolecules. *Methods Enzymol* **487**, 545-574 (2011).
20. N. Echols *et al.*, Graphical tools for macromolecular crystallography in PHENIX. *J Appl Crystallogr* **45**, 581-586 (2012).
21. P. V. Afonine *et al.*, Real-space refinement in PHENIX for cryo-EM and crystallography. *Acta Crystallogr D Struct Biol* **74**, 531-544 (2018).
22. S. H. Scheres, Processing of Structurally Heterogeneous Cryo-EM Data in RELION. *Methods Enzymol* **579**, 125-157 (2016).

23. N. Jullien, J. P. Herman, LUEGO: a cost and time saving gel shift procedure. *Biotechniques* **51**, 267-269 (2011).
24. R. C. Edgar, MUSCLE: multiple sequence alignment with high accuracy and high throughput. *Nucleic Acids Res* **32**, 1792-1797 (2004).
25. F. Madeira *et al.*, The EMBL-EBI search and sequence analysis tools APIs in 2019. *Nucleic Acids Res* **47**, W636-W641 (2019).
26. C. J. Williams *et al.*, MolProbity: More and better reference data for improved all-atom structure validation. *Protein Sci* **27**, 293-315 (2018).
27. P. V. Afonine *et al.*, New tools for the analysis and validation of cryo-EM maps and atomic models. *Acta Crystallogr D Struct Biol* **74**, 814-840 (2018).
28. B. A. Barad *et al.*, EMRinger: side chain-directed model and map validation for 3D cryo-electron microscopy. *Nat Methods* **12**, 943-946 (2015).
29. Y. Z. Tan *et al.*, Addressing preferred specimen orientation in single-particle cryo-EM through tilting. *Nat Methods* **14**, 793-796 (2017).
30. J. Pei, B. H. Kim, N. V. Grishin, PROMALS3D: a tool for multiple protein sequence and structure alignments. *Nucleic Acids Res* **36**, 2295-2300 (2008).
31. N. Mizuno *et al.*, MuB is an AAA+ ATPase that forms helical filaments to control target selection for DNA transposition. *Proc. Natl. Acad. Sci. U. S. A.* **110**, E2441-2450 (2013).
